# Integrating genomics and transcriptomics to identify candidate genes for high egg production in Wulong geese (Anser cygnoides orientalis)

**DOI:** 10.1186/s12864-023-09603-y

**Published:** 2023-08-24

**Authors:** Jingjing Liu, Yu Xiao, Pengwei Ren, Shuer Zhang, Yang Liu, Mingxia Zhu

**Affiliations:** 1 College of Agronomy and Agricultural Engineering, Liaocheng University, Liaocheng, 252000 China; 2 Shandong Animal Husbandry General Station, Jinan, 250010 China

**Keywords:** Wulong geese, High egg production trait, Whole-genome resequencing, Transcriptome sequencing

## Abstract

**Background:**

Wulong geese (*Anser cygnoides orientalis*) are known for their excellent egg-laying performance. However, they show considerable population differences in egg-laying behavior. This study combined genome-wide selection signal analysis with transcriptome analysis (RNA-seq) to identify the genes related to high egg production in Wulong geese.

**Results:**

A total of 132 selected genomic regions were screened using genome-wide selection signal analysis, and 130 genes related to high egg production were annotated in these regions. These selected genes were enriched in pathways related to egg production, including oocyte meiosis, the estrogen signaling pathway, the oxytocin signaling pathway, and progesterone-mediated oocyte maturation. Furthermore, a total of 890 differentially expressed genes (DEGs), including 340 up-regulated and 550 down-regulated genes, were identified by RNA-seq. Two genes — *GCG* and *FAP* — were common to the list of selected genes and DEGs. A non-synonymous single nucleotide polymorphism was identified in an exon of *FAP*.

**Conclusions:**

Based on genome-wide selection signal analysis and transcriptome data, *GCG* and *FAP* were identified as candidate genes associated with high egg production in Wulong geese. These findings could promote the breeding of Wulong geese with high egg production abilities and provide a theoretical basis for exploring the mechanisms of reproductive regulation in poultry.

**Supplementary Information:**

The online version contains supplementary material available at 10.1186/s12864-023-09603-y.

## Introduction

Wulong geese are among the most prolific egg-producing breeds of geese worldwide. However, they show significant population differences in egg-laying performance, with high-yielding geese (HYG) producing 120–140 eggs and low-yielding geese (LYG) producing 70–80 eggs annually. Therefore, the breeding of Wulong geese capable of producing a large number of eggs is an important objective in the field of goose farming. However, egg-laying performance is a low-heritability trait in geese [1]. Hence, traditional breeding methods have been ineffective in improving the egg-laying performance of geese. With the development of second-generation sequencing technology and the gradual reduction of sequencing costs, new opportunities to improve quantitative traits with low heritability have emerged.

Genome-wide selection signal analysis is an effective tool for identifying candidate genes related to egg-laying performance. Zhang et al. [2] detected selection signatures in Dwarf Brown-egg Layers and Silky Fowl chickens, identifying potential genes related to growth, reproduction, egg laying, and immune response (*GRHL3*, *CDK1*, *AKT1*, and *KMD3A*). Liu et al. [3] performed genome-wide selection signal analysis in four goose breeds (Lion-head, Zhedong White, Taihu, and Zi geese) and identified candidate genes and pathways associated with egg production, providing key insights into the history of artificial selection among local geese in China.

Ovaries are an important organ for egg production in poultry. Therefore, ovarian transcriptome sequencing is widely used to study egg-laying performance in birds. Zhang et al. [4] compared the ovarian transcriptomes of Lingyun Black-Bone chickens with high and low rates of egg production. They identified key candidate genes associated with the rate of egg production and demonstrated that longevity-regulating pathways and the multispecies signaling pathway, estrogen signaling pathway, and PPAR signaling pathway play important roles in regulating egg-laying rates in these birds. Zhu et al. [5] analyzed the transcriptomes of granulosa cells from chicken ovarian follicles at different stages and identified many cell signaling genes (*AMH*, inhibin, activin, and *BMP*) and transcription factors (*SMAD3*, *SMAD5*, *ID1*, *ID2*, and *ID3*) involved in follicular development. Zhang et al. [6] analyzed and compared ovarian mRNA levels between Jinghai Yellow chickens with high and low egg yields, identifying five candidate genes associated with egg production (*ZP2*, *WNT4*, *AMH*, *IGF1*, and *CYP17A1*).

Although previous studies have successfully uncovered key signaling pathways and candidate genes associated with egg-laying performance, the regulatory mechanisms underlying egg production in poultry remain to be fully elucidated. To our knowledge, few studies have integrated genome and transcriptome analyses to identify the major genes and signaling pathways associated with egg production, especially in geese. Therefore, to provide a theoretical basis for the breeding of Wulong geese with good egg-laying performance and accelerate the breeding process, we conducted genome-wide resequencing and transcriptome sequencing in Wulong geese with different egg yields and identified candidate genes associated with good egg-laying performance.

## Results

### Whole-genome sequencing and alignment

Table 1 shows the results of whole-genome resequencing and the alignment of gene sequences from HYG and LYG to the goose reference genome (AnsCyg_PRJNA183603_v1.0). A total of 2970.94 G raw reads were obtained after sequencing. After filtering, 2945.03 G clean reads were obtained. The average Q20 and Q30 values were 98.06% and 91.25%, respectively, and the average GC content was 42.46%. After quality control and filtering, the sequencing data were aligned to the reference genome, and the average alignment rate was 98.06%.

**Table 1:** Genome sequencing data and coverage statistics

Species	Raw reads	Clean reads	Q20/%	Q30/%	GC content/%	Mapping rate/%
Wulong geese (*Anser cygnoides orientalis*)	2 970.94 Gb	2 945.03 Gb	96.55	91.25	42.46	98.06

### Single nucleotide polymorphism (SNP) detection

As shown in Table 2, a total of 17,921,217 SNPs were detected in Wulong geese. Annotation analysis revealed that the proportion of SNPs located in intergenic regions, introns, and exons was 49.59%, 44.92%, and 1.58%, respectively. Notably, there were 128,988 non-synonymous mutations in the exonic regions. Moreover, there were 11,962,681 transitions (Ts) and 6,152,233 transversions (Tv), resulting in a Ts/Tv ratio of 1.9444.

**Table 2:** Classification of single nucleotide polymorphisms (SNPs)

Type of SNP		No. of SNPs/% of sites
Exon	Mutation leading to a stop codon	1532
	Mutation leading to the loss of a stop codon	268
	Synonymous mutation	151,954
	Non-synonymous mutation	128,988
Intron		8,050,248
Splice sites		4191
Upstream of gene		236,969
Downstream of gene		225,071
Upstream of one gene but downstream of another gene		12,766
Inter-genic region		8,887,190
Percentage of homozygous sites (of all genotypic sites)		98.93%
Percentage of heterozygous sites (of all genotypic sites)		1.07%
Transitions		11,962,681
Transversions		6,152,233
Transition/Transversion ratio		1.9444
Total number of sites		17,921,217

### Selection signal analysis

**Fig. 1:**
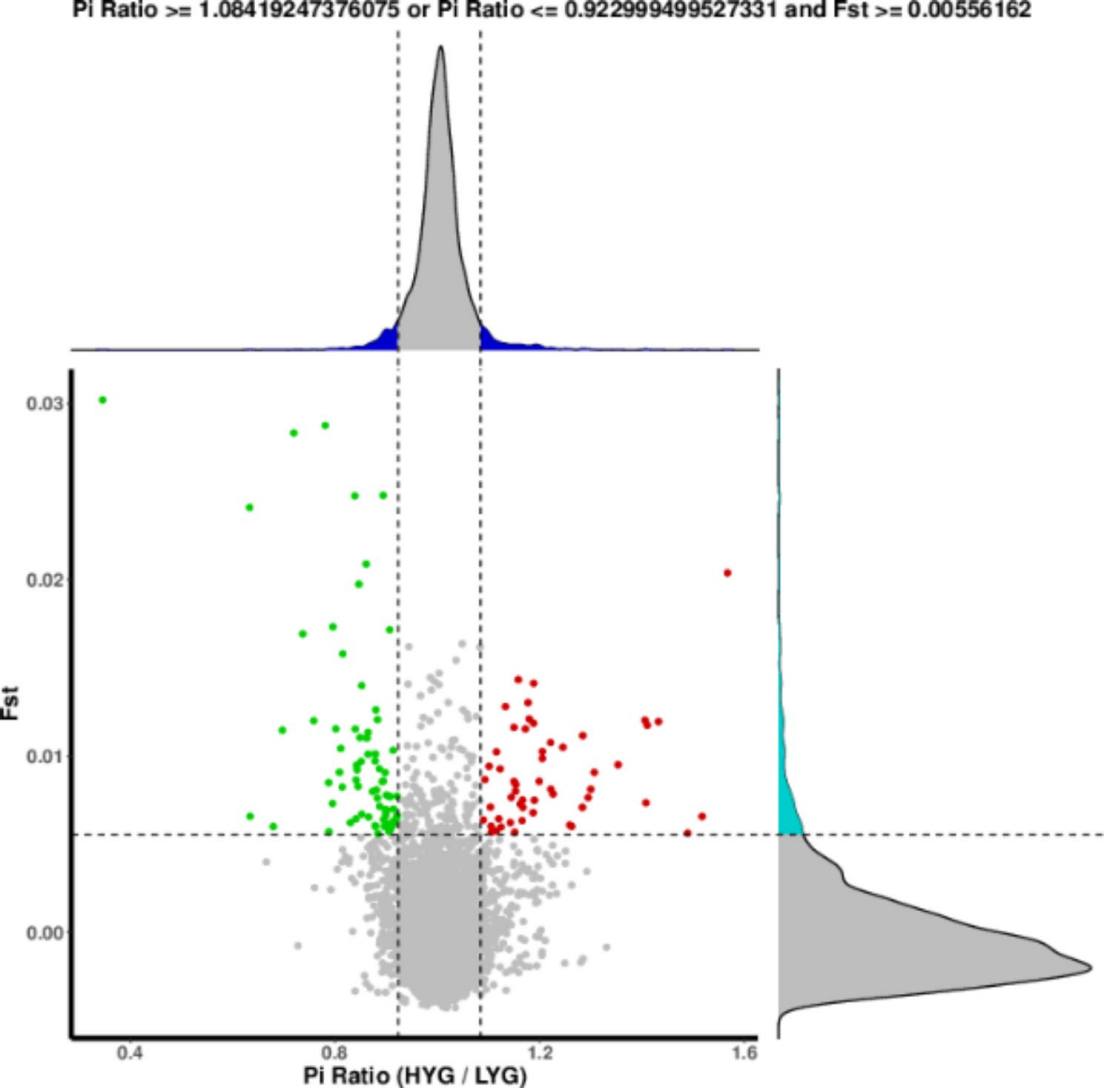
Genome-wide selection analysis based on the Fst and Pi ratio. The red dots in the figure indicate the areas where HYG are selected, and the green dots indicate the areas where LYG are selected. The curves in the top graph indicate the frequency of distribution of Pi Ratio values, and the curves in the right graph indicate the frequency of distribution of Fst values. The dashed line in the graph indicates the 95% confidence interval

Selection analysis was performed based on the fixation index (Fst) and Pi ratio (Fig. 1). With the threshold set to the top 5%, a total of 132 candidate regions under selection were identified, and 130 genes were annotated in these regions. Furthermore, 363 SNPs were annotated in the exonic regions of 57 genes (Table 3). The relevant information has been added to Supplementary Table 1.

**Table 3:** Genes annotated for non-synonymous mutation sites in exonic regions

Chromosome	Genes
NW_013185654.1	*AP3M1, CCAR1, HPS6, NCOA4, NDST2, PLAU, STOX1, UNC5B, USP54*
NW_013185655.1	*ANAPC4, EFCAB11, KCNK13, PI4K2B, SOD3, SPON2, TACC3, TDP1, TMEM251, UBR7, ZCCHC4*
NW_013185657.1	*ARFGAP2, ARHGAP1, ATG13, CAPN3, CKAP5, GANC, HARBI1, MGA, PACSIN3, TMEM87A, VPS39, ZNF408*
NW_013185658.1	*RPAP3, XRCC6BP1*
NW_013185659.1	*EFCAB2, KIF26B*
NW_013185660.1	*DPP4, FAP, IFIH1*
NW_013185661.1	*ABCB5, ITGB8, MACC1, OTUD1, TWISTNB*
NW_013185662.1	*FAM110C, TINAG*
NW_013185664.1	*E2F6, RASL11B*
NW_013185666.1	*PPM1F*
NW_013185668.1	*NPAS3*
NW_013185669.1	*VTI1A*
NW_013185670.1	*ACTB, FBXL18, FSCN1*
NW_013185671.1	*SPOCK3*
NW_013185673.1	*COQ3, PNISR*

### Gene Ontology (GO) and Kyoto Encyclopedia of Genes and Genomes (KEGG) analysis of selected region-specific genes

**Fig. 2:**
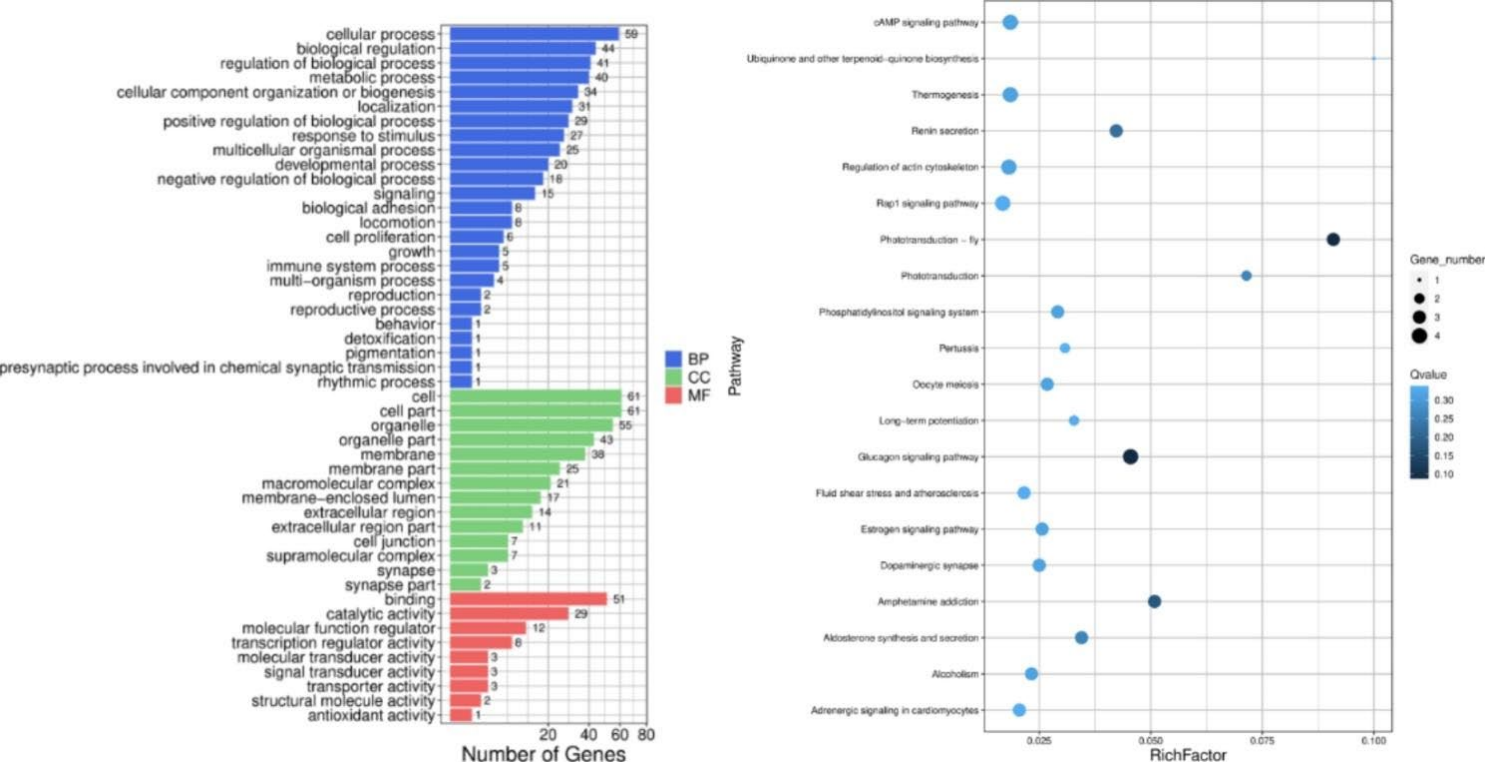
Functional enrichment results for the genes under selection. (**A**) GO enrichment analysis of genes in the selected region. (**B**) KEGG pathway enrichment analysis of genes in the selected region

The 130 selected genes were subjected to GO and KEGG enrichment analyses (Fig. 2). There were 48 enriched GO terms, including 25 terms under the biological processes module, 14 under the cellular components module, and 9 under the molecular functions module. In addition, 130 KEGG pathways were enriched, among which 19 pathways were significantly enriched (*P* < 0.05). Table 4 shows the GO terms and KEGG pathways enriched for the genes under selection.

**Table 4:** Genes related to egg-laying behavior in Wulong geese

GO term/KEGG Pathway	Number of genes	Genes
Reproduction	2	*ITGB8*, *NCOA4*
Reproductive process	2	*ITGB8*, *NCOA4*
Oocyte meiosis	3	*ANAPC4*, *CALM1*, *EFCAB2*
Estrogen signaling pathway	3	*CALM1*, *CREB5*, *EFCAB2*
Glucagon signaling pathway	4	*CALM1*, *CREB5*, *EFCAB2*, *GCG*

### Transcriptome sequencing and alignment analysis

As shown in Table 5, a total of 783,098,608 raw reads were obtained from 16 samples, and 775,617,642 clean reads were obtained after quality control. The Q20 values of all samples were above 97%, and the Q30 values were above 93%. Meanwhile, the proportion of reads mapped to the reference genome sequence for each sample was above 80%.

**Table 5:** Data quality control and sequence alignment statistics

Sample	Raw reads	Raw bases	Clean reads	Clean bases	Q20 (%)	Q30 (%)	Total mapped	GC content (%)
LYG1	48,894,902	7,383,130,202	48,438,856	7,173,692,030	97.98	94.24	40,828,110 (84.29%)	49.34
LYG2	51,603,238	7,792,088,938	51,142,864	7,569,358,653	98.04	94.35	42,857,812 (83.8%)	49.83
LYG3	51,511,510	7,778,238,010	50,930,658	7,458,103,284	98.02	94.35	42,111,773 (82.68%)	50.79
LYG4	46,951,716	7,089,709,116	46,529,092	6,913,704,737	97.98	94.27	38,440,527 (82.62%)	49.67
LYG5	48,802,436	7,369,167,836	48,341,000	7,173,190,445	97.85	93.89	41,206,994 (85.24%)	49.32
LYG6	45,409,546	6,856,841,446	45,001,650	6,659,690,342	98	94.3	38,072,950 (84.6%)	50.09
LYG7	57,057,622	8,615,700,922	56,550,002	8,267,490,008	98.13	94.64	46,726,144 (82.63%)	50.34
LYG8	49,414,826	7,461,638,726	49,001,222	7,226,360,701	98.1	94.53	40,686,970 (83.03%)	49.82
HYG1	50,323,602	7,598,863,902	49,896,922	7,370,435,258	98.2	94.76	41,913,290 (84.00%)	49.65
HYG2	52,026,146	7,855,948,046	51,448,188	7,545,110,325	98.07	94.52	41,770,795 (81.19%)	51.72
HYG3	39,860,598	6,018,950,298	39,525,368	5,891,888,669	98.03	94.33	33,208,251 (84.02%)	48.26
HYG4	49,255,666	7,437,605,566	48,702,710	7,167,698,776	97.81	93.88	39,283,208 (80.66%)	50.93
HYG5	44,658,944	6,743,500,544	44,213,396	6,571,608,752	97.94	94.19	35,927,691 (81.26%)	49.80
HYG6	47,326,222	7,146,259,522	46,950,460	6,963,852,732	98.15	94.65	38,867,239 (82.78%)	49.73
HYG7	48,458,600	7,317,248,600	47,897,162	7,042,938,629	97.85	94	38,900,039 (81.22%)	50.47
HYG8	51,543,034	7,782,998,134	51,048,092	7,505,637,591	98	94.31	41,616,680 (81.52%)	50.69

### Gene annotation and differential gene expression analysis

**Fig. 3:**
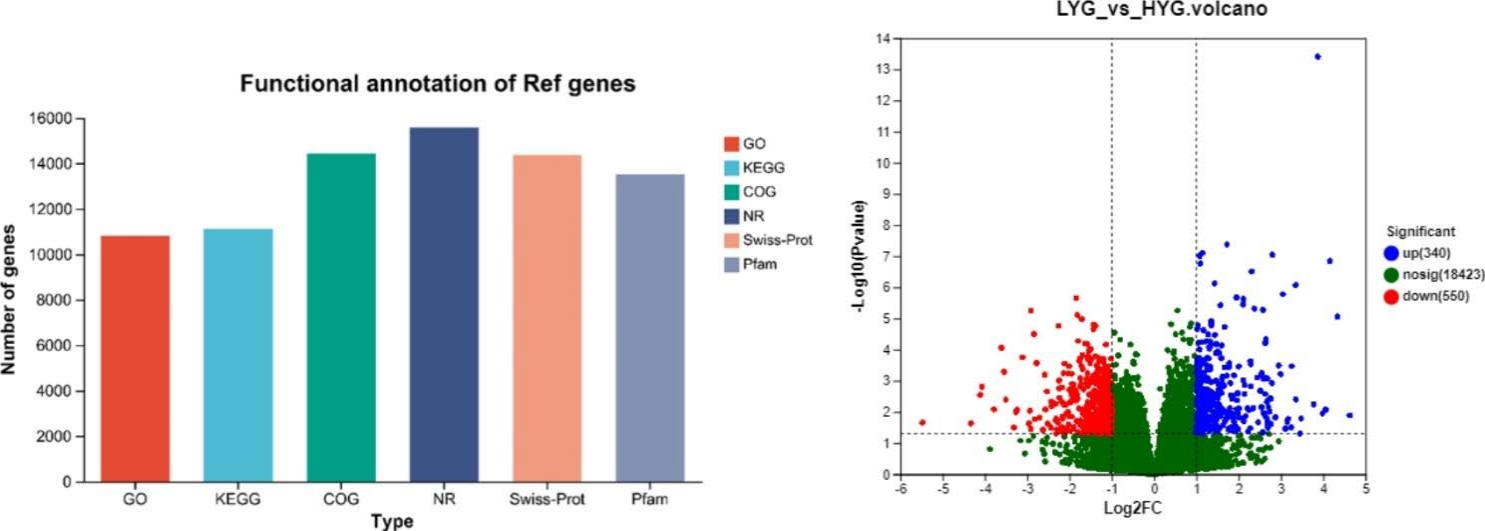
Annotated genes and differentially expressed genes. (**A**) Annotated genes. (**B**) Volcano plot of differentially expressed genes

As shown in Fig. 3, a total of 20,868 genes were annotated after RNA-seq. Their expression was quantified, and differential expression analysis was performed. Subsequently, a total of 890 differentially expressed genes (DEGs) were detected. Among these DEGs, 340 were up-regulated and 550 were down-regulated in HYG.

### Functional annotation and enrichment analysis of DEGs

**Fig. 4:**
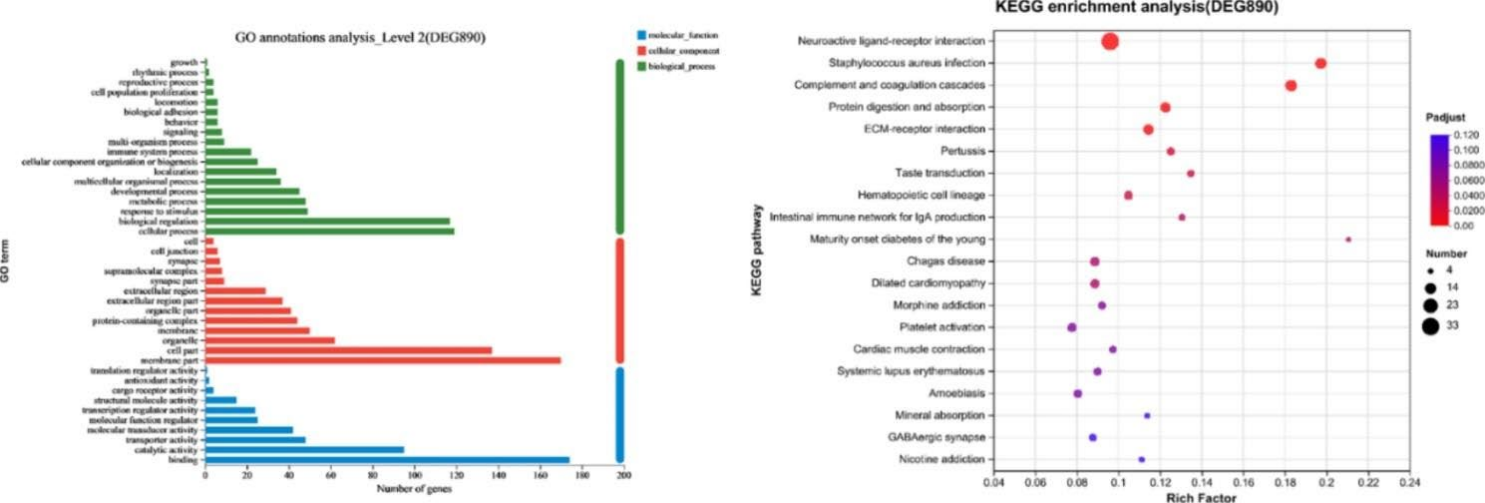
Functional annotation and enrichment of differentially expressed genes. (**A**) GO functional annotation of differently expressed genes. (**B**) KEGG pathway enrichment analysis of differently expressed genes

The DEGs were subjected to GO functional annotation and KEGG pathway enrichment analysis (Fig. 4). A total of 41 GO terms were annotated, including 18 terms under the biological processes module, 13 under the cellular components module, and 10 under the molecular functions module. Further, 243 KEGG pathways were enriched, of which 42 were significantly enriched. The GO terms and KEGG pathways related to egg production are shown in Table 6.

**Table 6:** Genes related to the egg-laying characteristics of Wulong geese

GO term/KEGG Pathway	Number of genes	Genes
Reproductive process	3	*GTSF1*, *HOXD10*, *TH*
Oxytocin signaling pathway	5	*CACNG5*, *FGF8623*, *FGF8586*, *GNAO1*, *MYL9*
Gonadotropin hormone-releasing hormone secretion	1	*TRPC5*
Estrogen signaling pathway	2	*GNAO1*, *CREB3L1*
Prolactin signaling pathway	1	*TH*

### DEGs under selection in ovarian tissue

The overlap between DEGs (obtained from transcriptome sequencing) and genes under selection (identified by genome-wide selection signal analysis) was examined. Two genes, *GCG* and *FAP*, were found to be common between both lists. Additionally, a non-synonymous SNP was detected within an exon of the *FAP* gene (Table 7).

**Table 7:** Differentially expressed genes under selection in ovarian tissue

Gene name	Log2FC (HYG/LYG)	*P* value	Regulation	Exonic nonsynonymous SNP
*GCG*	1.98	0.01	Up-regulated	None
*FAP*	1.08	0.00	Up-regulated	NW_013185660.1 C > A

### Quantitative real-time PCR (qRT-PCR) validation of DEGs

**Fig. 5:**
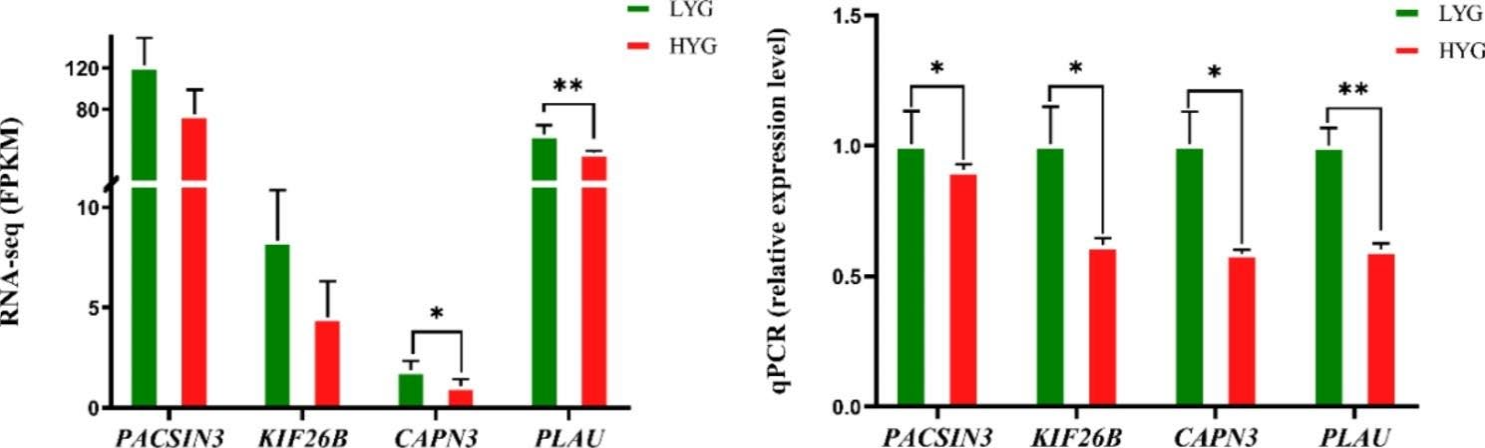
Expression of genes associated with egg-laying traits in Wulong geese **A**) Gene expression examined using RNA-seq. **B**) Gene expression examined using qRT-PCR. “**” indicates extremely significant differences at the 0.01 level, and “*” indicates significant differences at the 0.05 level. HYG: high-yielding geese; LYG, low-yielding geese

Four DEGs were randomly selected for the qRT-PCR validation of sequencing results. As shown in Fig. 5, the qRT-PCR results were consistent with RNA-seq findings.

## Discussion

Whole-genome resequencing is a high-throughput sequencing technology that can be used to sequence the entire genome of a species and discover new genomic variations [7]. Selection signal analysis is a tool used to uncover evidence of natural selection in certain genes or regions via the statistical analysis of genomic data [8]. There are various methods for selection signal analysis, and they can be categorized into four types based on their primary principles [9]: (i) population differentiation (such as Fst-Wright’s [10] and π [11]), (ii) local variation in genomic regions (such as ROH [12]), (iii) allele frequency spectra (such as Tajima’s D [13], Fay and Wu’s H [14], CL [15] R, and ZH [16] P), and (iv) linkage disequilibrium (such as iHS [17] and EHH [18]).

In this study, we conducted whole-genome resequencing at a depth of 10× using samples from a total of 249 high and low-yield Wulong geese, obtaining 2945.03 G of high-quality reads. The effective reads were aligned to the goose reference genome, and the average alignment rate was 98.06%, indicating the high quality and reliability of the sequencing results. A total of 17,921,217 SNP loci were detected in the population of Wulong geese. So far, there has been limited research on the genomic characteristics of Wulong geese. However, the rich data obtained in the present study provide a solid foundation for future genetic studies on this breed.

In this study, a total of 130 genes were obtained from the genome-wide selection signal analysis and subjected to GO and KEGG enrichment analysis. Reproduction, reproductive processes, oocyte meiosis, the estrogen signaling pathway, and the glucagon signaling pathway were found to be related to egg production in Wulong geese. The genes enriched in these pathways included *ITGB8*, *NCOA4*, *ANAPC4*, *CALM1*, *EFCAB2*, *CREB5*, and *GCG*. *CALM1* is an important regulator of testosterone production in chicken follicular cells and is closely related to reproductive performance in poultry [19, 20].

Transcriptomics refers to the study of gene expression and variations at the RNA level and reveals temporal and spatial differences in gene expression across various tissues and organs in different animals [21, 22]. As sequencing has become more cost-friendly, transcriptomics has gradually become the preferred tool for studying differential gene expression. It has several advantages, including high throughput, high coverage, wide applicability, low false positive rates, and high reproducibility, and it has been widely applied in scientific research [23]. Transcriptomic technology can be used to accurately evaluate the expression levels of all genes in specific tissues or cells and can also help in detecting new and rare transcripts [24, 25].

Our study focused on Wulong geese with high and low egg yields. Given that the ovaries are important for egg production, and ovarian development, follicular development, and ovulation are all crucial for this process [26], we collected ovarian tissue from Wulong geese and performed high-throughput RNA-seq using Illumina NovaSeq 6000 technology. A total of 775,617,642 high-quality reads were obtained, and the filtered reads were compared with the goose reference genome, providing a comparison rate of over 80%. Hence, a transcriptome library for Wulong geese was successfully constructed, and the sequencing quality was excellent, providing a large amount of genetic data for research on egg-laying performance in geese. The obtained data were annotated to specific genes and subjected to differential gene expression analysis, resulting in the identification of 890 DEGs. GO functional annotation and KEGG pathway enrichment analysis were performed on these DEGs. Reproductive processes, the oxytocin signaling pathway, GnRH secretion, the estrogen signaling pathway, and the prolactin signaling pathway were found to be related to egg-laying performance in Wulong geese. The genes involved included *GTSF1*, *HOXD10*, *TH*, *CACNG5*, *FGF8623*, *FGF8586*, *GNAO1*, *MYL9*, *TRPC5*, and *CREB3L1*. Among them, *CREB3L1* has been previously linked to ovarian development before and after egg-laying in Muscovy ducks [27].

Finally, a comprehensive analysis of data from whole-genome resequencing and transcriptome sequencing was performed. The comparison revealed two overlapping genes — *GCG* and *FAP*. Among them, *GCG* showed significantly higher expression levels in HYG than in LYG. Meanwhile, the expression of the *FAP* gene was also much higher in HYG. A non-synonymous mutation site was identified within an exon of the *FAP* gene. This candidate SNP may be associated with egg production traits in Wulong geese.

*GCG* encodes a hormone secreted by pancreatic alpha cells. This hormone can increase blood glucose levels, maintain energy metabolism, and stimulate glycogenolysis, gluconeogenesis, and lipolysis in the liver [28]. *FAP* encodes a serine protease belonging to the S9B prolyl oligopeptidase subfamily [29]. Studies report that *FAP* is involved in glucose and lipid metabolism and can regulate FGF-21 levels to improve glucose and lipid metabolism [30, 31]. In poultry, the process of egg production is energy- and nutrient-intensive [32], and glucose and fat are important sources of energy. Further, glucose is required for cell proliferation and estrogen synthesis [33], and moderate fat deposition helps in maintaining egg production and synthesizing the precursors of egg yolk [34, 35]. Therefore, both glucose and fat play an important role in the process of egg production. A previous study showed that the serum levels of glucose and triacyl glyceride are significantly higher in high-yield Wulong geese than in low-yield geese (*P* < 0.01) [36]. The results of our sequencing study show that *GCG* and *FAP* are highly expressed in high-yield geese, consistent with these previous findings. Therefore, we speculate that *GCG* and *FAP* may be involved in the positive regulation of egg production in Wulong geese, and their functions need to be explored further.

## Conclusion

In this study, genome-wide selection signal analysis was performed in high- and low-yield Wulong geese populations, and a total of 132 candidate regions accounting for 130 genes under selection were identified. Subsequently, transcriptome sequencing was performed, and 890 DEGs were identified. Interestingly, two genes — *GCG* and *FAP* — were up-regulated in high-yield Wulong geese, suggesting their involvement in the positive regulation of egg production. Additionally, a non-synonymous mutation site was detected in an exon of the *FAP* gene, representing a candidate SNP for the egg production trait in Wulong geese.

## Materials and methods

### Experimental samples

A total of 136 high egg-yielding (HYG; annual egg production, > 120 ± 10 eggs) and 113 low egg-yielding (LYG; annual egg production, < 80 ± 8 eggs) Wulong geese were selected. The geese (479 days old) were reared under the same environmental conditions and feeding protocols. The HYG had a wider pubic bone space, a fuller belly, and a shorter and thinner neck than the LYG. For DNA extraction, 2 mL of blood was collected from the wing vein of each goose and placed in an EDTA blood collection tube that was subsequently stored at -20 °C. Simultaneously, 16 geese were randomly selected from this group (8 high-yielding geese and 8 low-yielding geese) and sacrificed. Their ovarian tissue was collected and preserved in liquid nitrogen for RNA extraction. All experimental animals were provided by Shandong Wulong Geese Technology Development Co., Ltd. These geese(479 days old) were reared under the same nutritional level, environmental and feeding management conditions, and the HYG had a larger pubic bone spacing, a fuller belly, and a shorter and thinner neck compared to the LYG. The experimental protocol was approved by the Ethics Committee of Research at Liaocheng University (No. 2,003,041,001).

### DNA extraction and resequencing

DNA was extracted from the blood samples of Wulong geese using the TianGen Blood Genomic DNA Extraction Kit based on the manufacturer’s instructions. The integrity of the DNA was checked using 1% agarose gel electrophoresis, and DNA concentration was measured using a spectrophotometer. DNA samples that met quality and concentration requirements were sent to BGI Genomics for library construction and sequencing, with a sequencing depth of 10× per sample.

### SNP detection

Raw reads were processed using SOAPnuke (SOAPnuke1.5.6) software [37] for quality control. Reads containing adapters, excessive Ns, or low-quality bases were excluded using the following filtering parameters: SOAPnuke filter-n 0.01-l 20-q 0.5 --qualSys 2-G. Filtered and clean reads were aligned to the goose reference genome (AnsCyg_PRJNA183603_v1.0) using the BWA software [38], generating a SAM-format alignment file. Then, the samtools software [39] was used to convert the SAM file to a sorted BAM file, and fixmate and markdup operations were also performed. Finally, Qualimap2 (Version: 2.2.2-dev) [40] was used to perform quality control and statistical analysis on the BAM file using the BamQC tool. SNP detection was performed using the GATK software [41], and quality control was performed at each variant site. The Annovar software [42] was used to annotate and statistically analyze the variant sites.

### Selection signal analysis

Selection signals were screened based on the population genetic differentiation index (Fst) and population nucleotide diversity (π or Pi). VCFtools (Version: 0.1.16) [43] was used to calculate Fst and Pi ratio values for different genomic regions in the two populations, with the window size set to 100 kb and step size set to 50 kb.

Analysis based on population nucleotide diversity: Nucleotide diversity (π) refers to the average differences at each nucleotide between two randomly selected DNA sequences within the same population. Artificially selected populations tend to have lower genetic diversity and smaller π values, whereas wild populations exhibit greater genetic diversity and larger π values. That is, the larger the genotypic diversity within a population, the larger is the π value. In this study, we divided the π value of the treatment group (HYG) by the π value of the control group (LYG) to calculate the Pi Ratio. The regions outside the 95% confidence interval of the Pi Ratio were considered as significantly different regions. The regions with Pi Ratio values above the 95% confidence interval were considered as the regions under selection in the HYG population, while the regions with Pi Ratio values below the 95% confidence interval were considered as the regions under selection in the LYG population.

Analysis based on fixation index:

The fixation index (Fst) is a measure of genetic distances and thus reflects the degree of population differentiation. The formula for calculating FST is as follows:

1 $${\rm Fst} = \frac{{\pi }_{\text{Between }}-{\pi }_{\text{Within }}}{{\pi }_{\text{Between }}}$$

PiBetween represents the difference between two individuals from different populations, and PiWithin represents the difference between two individuals from the same population.

The Fst values for the same region were calculated by comparing two populations, and the 95% confidence interval was used to identify the significantly differentiated regions. Fst values above the confidence interval indicated significant differentiation between the two populations. Hence, regions with Fst values above the confidence interval were considered to be under selection.

Analysis based on combined parameters:

The selected regions identified through Fst and πθ screening were merged. The merged windows were then analyzed to obtain position information, the number of genes within each window, and the corresponding gene locations.

### Gene annotation and functional enrichment analysis

Based on the goose genome database (AnsCyg_PRJNA183603_v1.0), the selected loci were annotated to specific genes. The enrichment software developed by BGI Genomics was used for the GO and KEGG [44–46] enrichment analysis of the genes in the selected regions.

### Transcriptome data comparison and expression analysis

Total RNA was extracted from ovarian tissues and checked for concentration and purity using Nanodrop2000. RNA integrity was verified using agarose gel electrophoresis. After all RNA samples passed quality control tests, they were stored on dry ice and sent to Shanghai Meiji Biomedical Technology Co., Ltd. for sequencing analysis using the Illumina Novaseq 6000 high-throughput sequencing platform. The raw sequencing data were filtered and quality-controlled using the fastp tool and then aligned to the goose reference genome using HISAT2 software [47]. The alignment results were subjected to quality control.

Gene expression levels were calculated using RSEM software [48], with FPKM values used as a measure of gene expression, as follows:

2 $${\text{F}\text{P}\text{K}\text{M}}_{i}=\frac{{X}_{i}}{\left(\frac{{\stackrel{\sim}{l}}_{i}}{{10}^{3}}\right)\left(\frac{N}{{10}^{6}}\right)}=\frac{{X}_{i}}{{\stackrel{\sim}{l}}_{i}N}\cdot {10}^{9}$$

Differential genes were screened using the DESeq2 software [49] based on the selection criteria of fold change ≥ 2 and *P <* 0.05.

### qRT-PCR

We used the Animal Tissue Total RNA extraction kit from TianGen Biotech Co., Ltd. to extract total RNA from Wulong geese ovaries. The RNA was reverse transcribed using the Prime Script™ RT reagent Kit from TaKaRa Biosciences. The reverse transcription system is described in Supplementary Table 2. The primer sequences (Supplementary Table 3) were designed using Primer 5.0 software and synthesized by Shanghai Sangon Biological Engineering Technology & Services Co., Ltd. qRT-PCR was performed on a CFX96 real-time system (Bio-Rad, Hercules, CA, USA); the reaction system and conditions are shown in Supplementary Tables 4 and 5. Three biological replicates and three technical replicates were used for each sample. The relative expression levels of each gene were calculated using the 2^−ΔΔCt^ method, with *GAPDH* as the reference gene. Gene expression bar charts were plotted using GraphPad Prism 7. Independent sample t-tests were performed using SPSS 26.0 statistical software.

### Electronic supplementary material

Below is the link to the electronic supplementary material.

Supplementary Material 1

## Data Availability

All data generated or analyzed during this study are included in this published article and its additional files, or in the following public repositories. Data have been submitted to a public database under the following accession numbers: whole genome re-sequencing data [PRJNA998587] (https://www.ncbi.nlm.nih.gov/bioproject/PRJNA998587) and transcriptome sequencing data [PRJNA977881] (https://www.ncbi.nlm.nih.gov/bioproject/PRJNA977881).

## Abbreviations

DEG: Differentially expressed gene
Fst: Fixation index
HYG: High-yielding geese
LYG: Low-yielding geese
