# Supplementary material for: Integrating genomics and transcriptomics to identify candidate genes for high egg production in Wulong geese (Anser cygnoides orientalis)
Source: BMC Genomics. 2023 Aug 24;24:481. doi: 10.1186/s12864-023-09603-y (PMC10464066; doi:10.1186/s12864-023-09603-y)
Supplement: Supplementary file 1 — Supplementary Material 1 [file 12864_2023_9603_MOESM1_ESM.docx]

# Supplementary Material

# Integrating genomics and transcriptomics to identify candidate genes for high egg production in Wulong geese (Anser cygnoides orientalis)

**Jingjing Liu^1^, Yu Xiao^1^, Pengwei Ren^1^, Shuer Zhang^2^,** **Liu Yang^1^, Mingxia Zhu^1,*^**

^1^College of Agronomy of Liaocheng University, Liaocheng 252000, China

^2^Shandong Animal Husbandry General Station, Jinan 250010, China

*Corresponding author.

E-mail address: zhumingxia@lcu.edu.cn

**Table S1** Nonsynonymous mutation sites

| Gene | CHR | Position | Position | REF | ALT | Amino acid variation |
| --- | --- | --- | --- | --- | --- | --- |
| *gene-ABCB5* | NW_013185661.1 | 1660007 | 1660007 | G | C | 1659931:exon1:c.G33C:p.K11N |
| *gene-HPS6* | NW_013185654.1 | 629289 | 629289 | G | C | 629261:exon1:c.G22C:p.A8P |
| *gene-HPS6* | NW_013185654.1 | 629289 | 629289 | G | T | 629261:exon1:c.G22T:p.A8S |
| *gene-HPS6* | NW_013185654.1 | 629298 | 629298 | G | C | 629261:exon1:c.G31C:p.A11P |
| *gene-HPS6* | NW_013185654.1 | 629389 | 629389 | T | G | 629261:exon1:c.T122G:p.V41G |
| *gene-HPS6* | NW_013185654.1 | 629406 | 629406 | G | A | 629261:exon1:c.G139A:p.E47K |
| *gene-HPS6* | NW_013185654.1 | 629410 | 629410 | C | T | 629261:exon1:c.C143T:p.S48L |
| *gene-HPS6* | NW_013185654.1 | 629599 | 629599 | G | A | 629261:exon1:c.G332A:p.R111Q |
| *gene-HPS6* | NW_013185654.1 | 629646 | 629646 | G | A | 629261:exon1:c.G379A:p.V127M |
| *gene-HPS6* | NW_013185654.1 | 629974 | 629974 | A | C | 629261:exon1:c.A707C:p.H236P |
| *gene-HPS6* | NW_013185654.1 | 630039 | 630039 | A | G | 629261:exon1:c.A772G:p.T258A |
| *gene-HPS6* | NW_013185654.1 | 630114 | 630114 | G | A | 629261:exon1:c.G847A:p.E283K |
| *gene-HPS6* | NW_013185654.1 | 630478 | 630478 | C | T | 629261:exon1:c.C1211T:p.A404V |
| *gene-HPS6* | NW_013185654.1 | 630677 | 630677 | C | G | 629261:exon1:c.C1410G:p.D470E |
| *gene-HPS6* | NW_013185654.1 | 630847 | 630847 | A | C | 629261:exon1:c.A1580C:p.H527P |
| *gene-HPS6* | NW_013185654.1 | 630869 | 630869 | C | G | 629261:exon1:c.C1602G:p.D534E |
| *gene-HPS6* | NW_013185654.1 | 630973 | 630973 | T | C | 629261:exon1:c.T1706C:p.L569S |
| *gene-HPS6* | NW_013185654.1 | 630993 | 630993 | A | C | 629261:exon1:c.A1726C:p.T576P |
| *gene-HPS6* | NW_013185654.1 | 631008 | 631008 | G | C | 629261:exon1:c.G1741C:p.A581P |
| *gene-HPS6* | NW_013185654.1 | 631018 | 631018 | G | C | 629261:exon1:c.G1751C:p.R584P |
| *gene-HPS6* | NW_013185654.1 | 631048 | 631048 | T | G | 629261:exon1:c.T1781G:p.V594G |
| *gene-HPS6* | NW_013185654.1 | 631054 | 631054 | A | G | 629261:exon1:c.A1787G:p.D596G |
| *gene-HPS6* | NW_013185654.1 | 631072 | 631072 | T | G | 629261:exon1:c.T1805G:p.V602G |
| *gene-HPS6* | NW_013185654.1 | 631102 | 631102 | T | G | 629261:exon1:c.T1835G:p.V612G |
| *gene-ACTB* | NW_013185670.1 | 7295898 | 7295898 | A | C | 7292612:exon1:c.T7G:p.F3V |
| *gene-TWISTNB* | NW_013185661.1 | 1316746 | 1316746 | T | C | 1315539:exon3:c.A476G:p.E159G |
| *gene-TWISTNB* | NW_013185661.1 | 1320109 | 1320109 | C | T | 1315539:exon1:c.G118A:p.V40I |
| *gene-ANAPC4* | NW_013185655.1 | 14403983 | 14403983 | A | G | 14387873:exon26:c.A1783G:p.I595V;14387873:exon26:c.A1783G:p.I595V |
| *gene-MACC1* | NW_013185661.1 | 1501662 | 1501662 | T | C | 1489209:exon3:c.A509G:p.Y170C |
| *gene-MACC1* | NW_013185661.1 | 1501780 | 1501780 | A | G | 1489209:exon3:c.T391C:p.S131P |
| *gene-MACC1* | NW_013185661.1 | 1504395 | 1504395 | T | A | 1489209:exon2:c.A22T:p.S8C |
| *gene-AP3M1* | NW_013185654.1 | 23172036 | 23172036 | A | T | 23167673:exon5:c.A642T:p.R214S |
| *gene-ITGB8* | NW_013185661.1 | 1606308 | 1606308 | C | A | 1589857:exon8:c.C1001A:p.P334H;1584530:exon9:c.C1256A:p.P419H;1584530:exon9:c.C1256A:p.P419H;1587311:exon9:c.C1118A:p.P373H |
| *gene-ITGB8* | NW_013185661.1 | 1608338 | 1608338 | A | G | 1589857:exon9:c.A1423G:p.T475A;1584530:exon10:c.A1678G:p.T560A;1584530:exon10:c.A1678G:p.T560A;1587311:exon10:c.A1540G:p.T514A |
| *gene-ARFGAP2* | NW_013185657.1 | 11002806 | 11002806 | G | A | 11001167:exon13:c.C1253T:p.A418V |
| *gene-ABCB5* | NW_013185661.1 | 1662611 | 1662611 | G | A | 1659931:exon4:c.G316A:p.V106M |
| *gene-ABCB5* | NW_013185661.1 | 1669086 | 1669086 | A | G | 1659931:exon10:c.A1181G:p.N394S |
| *gene-ABCB5* | NW_013185661.1 | 1677230 | 1677230 | T | C | 1659931:exon15:c.T1886C:p.I629T |
| *gene-ABCB5* | NW_013185661.1 | 1684804 | 1684804 | G | A | 1659931:exon21:c.G2627A:p.R876Q |
| *gene-ARHGAP1* | NW_013185657.1 | 10737054 | 10737054 | T | C | 10732210:exon11:c.A994G:p.T332A;10732210:exon11:c.A994G:p.T332A |
| *gene-SPON2* | NW_013185655.1 | 2067435 | 2067435 | T | C | 2062291:exon4:c.A787G:p.I263V;2062292:exon5:c.A745G:p.I249V;2062293:exon5:c.A616G:p.I206V;2062293:exon5:c.A616G:p.I206V;2062293:exon6:c.A616G:p.I206V;2062293:exon6:c.A616G:p.I206V |
| *gene-SPON2* | NW_013185655.1 | 2069634 | 2069634 | A | G | 2062291:exon1:c.T41C:p.I14T |
| *gene-ATG13* | NW_013185657.1 | 10711947 | 10711947 | G | A | 10710467:exon3:c.G226A:p.V76I;10704820:exon4:c.G274A:p.V92I;10704820:exon4:c.G274A:p.V92I;10704820:exon4:c.G274A:p.V92I;10704820:exon4:c.G274A:p.V92I;10704820:exon4:c.G274A:p.V92I |
| *gene-TACC3* | NW_013185655.1 | 3131963 | 3131963 | T | C | 3128268:exon3:c.T535C:p.S179P |
| *gene-TACC3* | NW_013185655.1 | 3132141 | 3132141 | G | A | 3128268:exon3:c.G713A:p.R238K |
| *gene-TACC3* | NW_013185655.1 | 3132231 | 3132231 | C | T | 3128268:exon3:c.C803T:p.P268L |
| *gene-TACC3* | NW_013185655.1 | 3132380 | 3132380 | C | T | 3128268:exon3:c.C952T:p.P318S |
| *gene-TACC3* | NW_013185655.1 | 3132735 | 3132735 | C | A | 3128268:exon3:c.C1307A:p.P436Q |
| *gene-TACC3* | NW_013185655.1 | 3132798 | 3132798 | C | A | 3128268:exon3:c.C1370A:p.P457Q |
| *gene-TACC3* | NW_013185655.1 | 3134599 | 3134599 | C | T | 3128268:exon5:c.C1478T:p.T493I |
| *gene-TACC3* | NW_013185655.1 | 3134623 | 3134623 | A | C | 3128268:exon5:c.A1502C:p.D501A |
| *gene-TACC3* | NW_013185655.1 | 3134632 | 3134632 | C | A | 3128268:exon5:c.C1511A:p.P504Q |
| *gene-TACC3* | NW_013185655.1 | 3134683 | 3134683 | C | T | 3128268:exon5:c.C1562T:p.A521V |
| *gene-TACC3* | NW_013185655.1 | 3134745 | 3134745 | A | G | 3128268:exon5:c.A1624G:p.T542A |
| *gene-TACC3* | NW_013185655.1 | 3134760 | 3134760 | T | C | 3128268:exon5:c.T1639C:p.F547L |
| *gene-TACC3* | NW_013185655.1 | 3136941 | 3136941 | T | C | 3128268:exon8:c.T1862C:p.L621P |
| *gene-TACC3* | NW_013185655.1 | 3137896 | 3137896 | G | A | 3128268:exon9:c.G2107A:p.G703R |
| *gene-TACC3* | NW_013185655.1 | 3141138 | 3141138 | G | T | 3128268:exon10:c.G2121T:p.E707D |
| *gene-CAPN3* | NW_013185657.1 | 8329494 | 8329494 | C | T | 8323889:exon10:c.G614A:p.R205H;8323895:exon14:c.G1109A:p.R370H;8323897:exon18:c.G1694A:p.R565H;8323888:exon19:c.G2048A:p.R683H;8323872:exon21:c.G2168A:p.R723H |
| *gene-CCAR1* | NW_013185654.1 | 11591352 | 11591352 | C | T | 11590495:exon25:c.G3598A:p.V1200I |
| *gene-FAP* | NW_013185660.1 | 4377342 | 4377342 | C | T | 4335234:exon1:c.G23A:p.G8E |
| *gene-CKAP5* | NW_013185657.1 | 10785504 | 10785504 | G | T | 10780421:exon37:c.C5020A:p.Q1674K;10780421:exon38:c.C5131A:p.Q1711K;10778287:exon39:c.C5155A:p.Q1719K |
| *gene-IFIH1* | NW_013185660.1 | 4402971 | 4402971 | C | T | 4380717:exon4:c.G622A:p.D208N |
| *gene-IFIH1* | NW_013185660.1 | 4407333 | 4407333 | A | C | 4380717:exon2:c.T155G:p.V52G |
| *gene-IFIH1* | NW_013185660.1 | 4409282 | 4409282 | T | C | 4380717:exon1:c.A100G:p.I34V |
| *gene-IFIH1* | NW_013185660.1 | 4409288 | 4409288 | C | T | 4380717:exon1:c.G94A:p.D32N |
| *gene-COQ3* | NW_013185673.1 | 8497206 | 8497206 | C | T | 8497205:exon1:c.C1T:p.R1C |
| *gene-E2F6* | NW_013185664.1 | 5356659 | 5356659 | T | C | 5350168:exon1:c.A85G:p.T29A;5351659:exon1:c.A85G:p.T30A |
| *gene-DPP4* | NW_013185660.1 | 4281702 | 4281702 | C | T | 4272860:exon23:c.G1978A:p.G660S |
| *gene-EFCAB2* | NW_013185659.1 | 5757835 | 5757835 | A | G | 5757643:exon1:c.A14G:p.Y5C |
| *gene-EFCAB2* | NW_013185659.1 | 5757870 | 5757870 | G | T | 5757643:exon1:c.G49T:p.V17F |
| *gene-EFCAB2* | NW_013185659.1 | 5757883 | 5757883 | G | A | 5757643:exon1:c.G62A:p.R21H;5757274:exon2:c.G23A:p.R9H |
| *gene-EFCAB2* | NW_013185659.1 | 5757922 | 5757922 | A | G | 5757643:exon1:c.A101G:p.K34R;5757274:exon2:c.A62G:p.K22R |
| *gene-EFCAB2* | NW_013185659.1 | 5786868 | 5786868 | A | G | 5757643:exon6:c.A457G:p.K153E;5773821:exon6:c.A388G:p.K130E;5736815:exon8:c.A508G:p.K170E;5736815:exon9:c.A508G:p.K170E;5736815:exon9:c.A508G:p.K170E;5736815:exon9:c.A487G:p.K163E;5736815:exon10:c.A508G:p.K170E |
| *gene-EFCAB2* | NW_013185659.1 | 5787987 | 5787987 | T | A | 5757643:exon7:c.T563A:p.L188H;5773821:exon7:c.T494A:p.L165H;5736815:exon9:c.T614A:p.L205H;5736815:exon10:c.T614A:p.L205H;5736815:exon10:c.T614A:p.L205H;5736815:exon10:c.T593A:p.L198H;5736815:exon11:c.T614A:p.L205H |
| *gene-E2F6* | NW_013185664.1 | 5356658 | 5356658 | G | A | 5350168:exon1:c.C86T:p.T29I;5351659:exon1:c.C86T:p.T30I |
| *gene-KIF26B* | NW_013185659.1 | 5902435 | 5902435 | T | C | 5810921:exon2:c.T146C:p.V49A |
| *gene-KIF26B* | NW_013185659.1 | 5932665 | 5932665 | C | A | 5810921:exon3:c.C665A:p.T222N |
| *gene-KIF26B* | NW_013185659.1 | 6050995 | 6050995 | G | C | 5810921:exon9:c.G1724C:p.S575T |
| *gene-KIF26B* | NW_013185659.1 | 6081100 | 6081100 | A | G | 5810921:exon11:c.A2599G:p.I867V |
| *gene-KIF26B* | NW_013185659.1 | 6081221 | 6081221 | A | G | 5810921:exon11:c.A2720G:p.N907S |
| *gene-KIF26B* | NW_013185659.1 | 6081452 | 6081452 | G | A | 5810921:exon11:c.G2951A:p.R984K |
| *gene-KIF26B* | NW_013185659.1 | 6082183 | 6082183 | G | A | 5810921:exon11:c.G3682A:p.G1228S |
| *gene-KIF26B* | NW_013185659.1 | 6082357 | 6082357 | G | A | 5810921:exon11:c.G3856A:p.A1286T |
| *gene-KIF26B* | NW_013185659.1 | 6083317 | 6083317 | T | C | 5810921:exon11:c.T4816C:p.S1606P |
| *gene-KIF26B* | NW_013185659.1 | 6083664 | 6083664 | G | C | 5810921:exon11:c.G5163C:p.E1721D |
| *gene-KIF26B* | NW_013185659.1 | 6083728 | 6083728 | C | T | 5810921:exon11:c.C5227T:p.R1743C |
| *gene-EFCAB11* | NW_013185655.1 | 20778888 | 20778888 | T | C | 20774062:exon3:c.T149C:p.M50T |
| *gene-FBXL18* | NW_013185670.1 | 7284849 | 7284849 | G | A | 7265286:exon3:c.C992T:p.A331V;7269334:exon3:c.C716T:p.A240V |
| *gene-FBXL18* | NW_013185670.1 | 7284870 | 7284870 | C | T | 7265286:exon3:c.G971A:p.R324H;7269334:exon3:c.G695A:p.R233H |
| *gene-FBXL18* | NW_013185670.1 | 7285138 | 7285138 | C | T | 7265286:exon3:c.G703A:p.A235T;7269334:exon3:c.G427A:p.A144T |
| *gene-FBXL18* | NW_013185670.1 | 7287296 | 7287296 | A | C | 7269334:exon1:c.T29G:p.V10G |
| *gene-FBXL18* | NW_013185670.1 | 7287308 | 7287308 | A | G | 7269334:exon1:c.T17C:p.L6P |
| *gene-FBXL18* | NW_013185670.1 | 7287735 | 7287735 | C | G | 7265286:exon1:c.G305C:p.S102T |
| *gene-FBXL18* | NW_013185670.1 | 7287879 | 7287879 | C | T | 7265286:exon1:c.G161A:p.R54Q |
| *gene-FBXL18* | NW_013185670.1 | 7287919 | 7287919 | G | A | 7265286:exon1:c.C121T:p.R41C |
| *gene-FBXL18* | NW_013185670.1 | 7287951 | 7287951 | G | A | 7265286:exon1:c.C89T:p.P30L |
| *gene-FBXL18* | NW_013185670.1 | 7288029 | 7288029 | T | C | 7265286:exon1:c.A11G:p.D4G |
| *gene-EFCAB2* | NW_013185659.1 | 5757822 | 5757822 | A | G | 5757643:exon1:c.A1G:p.M1V |
| *gene-FSCN1* | NW_013185670.1 | 7314790 | 7314790 | G | A | 7314788:exon1:c.G2A:p.G1D |
| *gene-FSCN1* | NW_013185670.1 | 7314796 | 7314796 | G | T | 7314788:exon1:c.G8T:p.R3L |
| *gene-FSCN1* | NW_013185670.1 | 7314834 | 7314834 | T | G | 7314788:exon1:c.T46G:p.W16G |
| *gene-FSCN1* | NW_013185670.1 | 7314940 | 7314940 | A | C | 7314788:exon1:c.A152C:p.D51A |
| *gene-FSCN1* | NW_013185670.1 | 7314948 | 7314948 | T | G | 7314788:exon1:c.T160G:p.W54G |
| *gene-FSCN1* | NW_013185670.1 | 7314969 | 7314969 | A | C | 7314788:exon1:c.A181C:p.T61P |
| *gene-FSCN1* | NW_013185670.1 | 7315012 | 7315012 | A | C | 7314788:exon1:c.A224C:p.D75A |
| *gene-FSCN1* | NW_013185670.1 | 7315015 | 7315015 | A | C | 7314788:exon1:c.A227C:p.H76P |
| *gene-FSCN1* | NW_013185670.1 | 7315033 | 7315033 | A | C | 7314788:exon1:c.A245C:p.D82A |
| *gene-FSCN1* | NW_013185670.1 | 7315036 | 7315036 | G | C | 7314788:exon1:c.G248C:p.G83A |
| *gene-FSCN1* | NW_013185670.1 | 7315126 | 7315126 | A | C | 7314788:exon1:c.A338C:p.Y113S |
| *gene-FSCN1* | NW_013185670.1 | 7321678 | 7321678 | G | A | 7314788:exon3:c.G697A:p.A233T |
| *gene-FSCN1* | NW_013185670.1 | 7321706 | 7321706 | A | G | 7314788:exon3:c.A725G:p.K242R |
| *gene-FSCN1* | NW_013185670.1 | 7321995 | 7321995 | C | G | 7314788:exon4:c.C804G:p.I268M |
| *gene-FSCN1* | NW_013185670.1 | 7321996 | 7321996 | A | C | 7314788:exon4:c.A805C:p.I269L |
| *gene-FSCN1* | NW_013185670.1 | 7321998 | 7321998 | C | G | 7314788:exon4:c.C807G:p.I269M |
| *gene-FSCN1* | NW_013185670.1 | 7322068 | 7322068 | T | G | 7314788:exon4:c.T877G:p.S293A |
| *gene-FSCN1* | NW_013185670.1 | 7322069 | 7322069 | C | T | 7314788:exon4:c.C878T:p.S293F |
| *gene-FAM110C* | NW_013185662.1 | 12822446 | 12822446 | C | T | 12817335:exon1:c.G457A:p.A153T |
| *gene-FAP* | NW_013185660.1 | 4362804 | 4362804 | C | A | 4335234:exon10:c.G841T:p.A281S;4337549:exon10:c.G766T:p.A257S |
| *gene-GANC* | NW_013185657.1 | 8357883 | 8357883 | G | A | 8355981:exon22:c.C2480T:p.T827M;8355981:exon23:c.C2663T:p.T888M;8355981:exon23:c.C2657T:p.T886M |
| *gene-GANC* | NW_013185657.1 | 8359310 | 8359310 | G | A | 8355981:exon20:c.C2302T:p.R768W;8355981:exon21:c.C2485T:p.R829W;8355981:exon21:c.C2479T:p.R827W;8355981:exon21:c.C2485T:p.R829W |
| *gene-GANC* | NW_013185657.1 | 8359381 | 8359381 | C | T | 8355981:exon20:c.G2231A:p.C744Y;8355981:exon21:c.G2414A:p.C805Y;8355981:exon21:c.G2408A:p.C803Y;8355981:exon21:c.G2414A:p.C805Y |
| *gene-GANC* | NW_013185657.1 | 8359382 | 8359382 | A | G | 8355981:exon20:c.T2230C:p.C744R;8355981:exon21:c.T2413C:p.C805R;8355981:exon21:c.T2407C:p.C803R;8355981:exon21:c.T2413C:p.C805R |
| *gene-GANC* | NW_013185657.1 | 8366050 | 8366050 | A | C | 8355981:exon11:c.T1294G:p.F432V;8355981:exon12:c.T1477G:p.F493V;8355981:exon12:c.T1477G:p.F493V;8355981:exon12:c.T1477G:p.F493V |
| *gene-GANC* | NW_013185657.1 | 8368450 | 8368450 | G | T | 8355981:exon9:c.C970A:p.H324N;8355981:exon10:c.C1153A:p.H385N;8355981:exon10:c.C1153A:p.H385N;8355981:exon10:c.C1153A:p.H385N |
| *gene-GANC* | NW_013185657.1 | 8368911 | 8368911 | T | C | 8355981:exon8:c.A835G:p.N279D;8355981:exon9:c.A1018G:p.N340D;8355981:exon9:c.A1018G:p.N340D;8355981:exon9:c.A1018G:p.N340D |
| *gene-GANC* | NW_013185657.1 | 8368949 | 8368949 | C | A | 8355981:exon8:c.G797T:p.S266I;8355981:exon9:c.G980T:p.S327I;8355981:exon9:c.G980T:p.S327I;8355981:exon9:c.G980T:p.S327I |
| *gene-GANC* | NW_013185657.1 | 8368989 | 8368989 | C | T | 8355981:exon8:c.G757A:p.V253M;8355981:exon9:c.G940A:p.V314M;8355981:exon9:c.G940A:p.V314M;8355981:exon9:c.G940A:p.V314M |
| *gene-GANC* | NW_013185657.1 | 8371734 | 8371734 | C | T | 8355981:exon5:c.G410A:p.S137N;8355981:exon6:c.G593A:p.S198N;8355981:exon6:c.G593A:p.S198N;8355981:exon6:c.G593A:p.S198N |
| *gene-FBXL18* | NW_013185670.1 | 7284322 | 7284322 | A | G | 7265286:exon3:c.T1519C:p.Y507H;7269334:exon3:c.T1243C:p.Y416H |
| *gene-GANC* | NW_013185657.1 | 8376378 | 8376378 | G | C | 8355981:exon2:c.C186G:p.D62E;8355981:exon2:c.C186G:p.D62E;8355981:exon2:c.C186G:p.D62E;8355981:exon2:c.C186G:p.D62E |
| *gene-TMEM87A* | NW_013185657.1 | 8396671 | 8396671 | C | T | 8379829:exon15:c.C1343T:p.P448L |
| *gene-TMEM87A* | NW_013185657.1 | 8396687 | 8396687 | A | T | 8379829:exon15:c.A1359T:p.E453D |
| *gene-HARBI1* | NW_013185657.1 | 10703635 | 10703635 | G | A | 10702971:exon2:c.C788T:p.T263M |
| *gene-PNISR* | NW_013185673.1 | 8484379 | 8484379 | G | A | 8477465:exon6:c.G910A:p.V304I;8468474:exon7:c.G910A:p.V305I |
| *gene-PNISR* | NW_013185673.1 | 8491232 | 8491232 | C | A | 8477465:exon10:c.C1814A:p.A605D;8468474:exon11:c.C1814A:p.A606D |
| *gene-PNISR* | NW_013185673.1 | 8491331 | 8491331 | G | A | 8477465:exon10:c.G1913A:p.R638H;8468474:exon11:c.G1913A:p.R639H |
| *gene-HPS6* | NW_013185654.1 | 629280 | 629280 | C | T | 629261:exon1:c.C13T:p.P5S |
| *gene-IFIH1* | NW_013185660.1 | 4398808 | 4398808 | G | A | 4380717:exon5:c.C779T:p.A260V |
| *gene-COQ3* | NW_013185673.1 | 8497224 | 8497224 | C | T | 8497205:exon1:c.C19T:p.R7C |
| *gene-COQ3* | NW_013185673.1 | 8497231 | 8497231 | C | T | 8497205:exon1:c.C26T:p.S9F |
| *gene-COQ3* | NW_013185673.1 | 8497302 | 8497302 | C | T | 8497205:exon1:c.C97T:p.R33C |
| *gene-COQ3* | NW_013185673.1 | 8497326 | 8497326 | A | G | 8497205:exon1:c.A121G:p.S41G |
| *gene-COQ3* | NW_013185673.1 | 8497339 | 8497339 | T | C | 8497205:exon1:c.T134C:p.V45A |
| *gene-COQ3* | NW_013185673.1 | 8497359 | 8497359 | A | G | 8497205:exon1:c.A154G:p.R52G |
| *gene-COQ3* | NW_013185673.1 | 8497360 | 8497360 | G | A | 8497205:exon1:c.G155A:p.R52K |
| *gene-COQ3* | NW_013185673.1 | 8497732 | 8497732 | C | T | 8497205:exon2:c.C170T:p.T57M |
| *gene-COQ3* | NW_013185673.1 | 8499464 | 8499464 | T | C | 8497205:exon4:c.T632C:p.I211T |
| *gene-ITGB8* | NW_013185661.1 | 1606176 | 1606176 | C | T | 1589857:exon8:c.C869T:p.A290V;1584530:exon9:c.C1124T:p.A375V;1584530:exon9:c.C1124T:p.A375V;1587311:exon9:c.C986T:p.A329V |
| *gene-TINAG* | NW_013185662.1 | 8501755 | 8501755 | C | A | 8501730:exon11:c.G1380T:p.K460N |
| *gene-COQ3* | NW_013185673.1 | 8502646 | 8502646 | G | A | 8497205:exon6:c.G974A:p.G325E |
| *gene-KCNK13* | NW_013185655.1 | 20656776 | 20656776 | T | C | 20652177:exon2:c.A631G:p.I211V |
| *gene-KIF26B* | NW_013185659.1 | 5811013 | 5811013 | T | C | 5810921:exon1:c.T92C:p.L31P |
| *gene-MACC1* | NW_013185661.1 | 1501459 | 1501459 | C | G | 1489209:exon3:c.G712C:p.V238L |
| *gene-TINAG* | NW_013185662.1 | 8523670 | 8523670 | G | T | 8501730:exon7:c.C964A:p.P322T |
| *gene-SPOCK3* | NW_013185671.1 | 8525573 | 8525573 | T | C | 8448114:exon2:c.A112G:p.T38A;8448114:exon3:c.A196G:p.T66A;8448113:exon5:c.A451G:p.T151A;8448113:exon6:c.A460G:p.T154A |
| *gene-TINAG* | NW_013185662.1 | 8540864 | 8540864 | T | C | 8501730:exon3:c.A505G:p.I169V |
| *gene-TINAG* | NW_013185662.1 | 8546588 | 8546588 | G | C | 8501730:exon1:c.C135G:p.D45E |
| *gene-SPOCK3* | NW_013185671.1 | 8557919 | 8557919 | A | C | 8448114:exon2:c.T29G:p.V10G;8448114:exon3:c.T125G:p.V42G;8448113:exon5:c.T293G:p.V98G |
| *gene-MGA* | NW_013185657.1 | 9037142 | 9037142 | A | G | 9033688:exon25:c.T9418C:p.S3140P |
| *gene-NCOA4* | NW_013185654.1 | 17021829 | 17021829 | G | A | 17014481:exon8:c.G1691A:p.S564N |
| *gene-MGA* | NW_013185657.1 | 9058255 | 9058255 | T | C | 9033688:exon18:c.A6691G:p.N2231D |
| *gene-MGA* | NW_013185657.1 | 9061891 | 9061891 | G | C | 9033688:exon15:c.C5051G:p.T1684R |
| *gene-MGA* | NW_013185657.1 | 9075305 | 9075305 | A | T | 9033688:exon5:c.T2207A:p.M736K |
| *gene-MGA* | NW_013185657.1 | 9080094 | 9080094 | A | G | 9033688:exon3:c.T2074C:p.S692P |
| *gene-MGA* | NW_013185657.1 | 9080634 | 9080634 | C | T | 9033688:exon2:c.G1876A:p.V626I |
| *gene-NDST2* | NW_013185654.1 | 23616054 | 23616054 | G | C | 23595035:exon12:c.G2373C:p.L791F |
| *gene-HARBI1* | NW_013185657.1 | 10704675 | 10704675 | C | G | 10702971:exon1:c.G369C:p.E123D |
| *gene-HARBI1* | NW_013185657.1 | 10704854 | 10704854 | A | G | 10702971:exon1:c.T190C:p.S64P |
| *gene-NPAS3* | NW_013185668.1 | 8579729 | 8579729 | A | G | 8438424:exon3:c.A586G:p.R196G |
| *gene-ATG13* | NW_013185657.1 | 10720644 | 10720644 | T | C | 10710467:exon10:c.T820C:p.S274P;10704820:exon10:c.T769C:p.S257P;10704820:exon12:c.T979C:p.S327P;10704820:exon12:c.T979C:p.S327P;10704820:exon13:c.T1078C:p.S360P;10704820:exon13:c.T1051C:p.S351P |
| *gene-OTUD1* | NW_013185661.1 | 11399913 | 11399913 | T | G | 11397677:exon2:c.A371C:p.Y124S |
| *gene-PACSIN3* | NW_013185657.1 | 11026568 | 11026568 | G | A | 11021099:exon5:c.C667T:p.R223C |
| *gene-ZNF408* | NW_013185657.1 | 10759191 | 10759191 | A | G | 10758467:exon1:c.A166G:p.K56E |
| *gene-ZNF408* | NW_013185657.1 | 10759200 | 10759200 | G | A | 10758467:exon1:c.G175A:p.E59K |
| *gene-ZNF408* | NW_013185657.1 | 10759252 | 10759252 | A | G | 10758467:exon1:c.A227G:p.Q76R |
| *gene-ZNF408* | NW_013185657.1 | 10759254 | 10759254 | C | G | 10758467:exon1:c.C229G:p.Q77E |
| *gene-ZNF408* | NW_013185657.1 | 10759506 | 10759506 | C | T | 10758467:exon1:c.C481T:p.R161W |
| *gene-ZNF408* | NW_013185657.1 | 10759854 | 10759854 | A | C | 10758467:exon1:c.A829C:p.T277P |
| *gene-ZNF408* | NW_013185657.1 | 10759887 | 10759887 | G | A | 10758467:exon1:c.G862A:p.G288S |
| *gene-ZNF408* | NW_013185657.1 | 10760122 | 10760122 | T | A | 10758467:exon1:c.T1097A:p.I366K |
| *gene-ZNF408* | NW_013185657.1 | 10760310 | 10760310 | A | G | 10758467:exon1:c.A1285G:p.T429A |
| *gene-ZNF408* | NW_013185657.1 | 10760373 | 10760373 | A | G | 10758467:exon1:c.A1348G:p.S450G |
| *gene-ZNF408* | NW_013185657.1 | 10760374 | 10760374 | G | C | 10758467:exon1:c.G1349C:p.S450T |
| *gene-ZNF408* | NW_013185657.1 | 10760587 | 10760587 | T | G | 10758467:exon1:c.T1562G:p.V521G |
| *gene-PI4K2B* | NW_013185655.1 | 14357192 | 14357192 | G | C | 14356254:exon1:c.G94C:p.V32L |
| *gene-CKAP5* | NW_013185657.1 | 10792774 | 10792774 | T | C | 10780421:exon29:c.A3535G:p.T1179A;10780421:exon29:c.A3535G:p.T1179A;10780421:exon30:c.A3646G:p.T1216A |
| *gene-CKAP5* | NW_013185657.1 | 10793943 | 10793943 | G | A | 10778287:exon28:c.C3470T:p.T1157M;10780421:exon28:c.C3470T:p.T1158M |
| *gene-CKAP5* | NW_013185657.1 | 10801595 | 10801595 | A | C | 10778287:exon23:c.T2807G:p.V936G;10780421:exon23:c.T2807G:p.V936G;10780421:exon23:c.T2807G:p.V936G |
| *gene-CKAP5* | NW_013185657.1 | 10801596 | 10801596 | C | T | 10778287:exon23:c.G2806A:p.V936I;10780421:exon23:c.G2806A:p.V936I;10780421:exon23:c.G2806A:p.V936I |
| *gene-CKAP5* | NW_013185657.1 | 10811346 | 10811346 | G | T | 10778287:exon10:c.C1174A:p.P392T;10780421:exon10:c.C1174A:p.P392T;10780421:exon10:c.C1174A:p.P392T |
| *gene-PLAU* | NW_013185654.1 | 23293394 | 23293394 | C | T | 23288577:exon7:c.G574A:p.V192I |
| *gene-UNC5B* | NW_013185654.1 | 10981597 | 10981597 | T | C | 10981425:exon1:c.T106C:p.C36R;10981425:exon1:c.T106C:p.C37R |
| *gene-UNC5B* | NW_013185654.1 | 10981598 | 10981598 | G | A | 10981425:exon1:c.G107A:p.C36Y;10981425:exon1:c.G107A:p.C37Y |
| *gene-UNC5B* | NW_013185654.1 | 10981670 | 10981670 | C | T | 10981425:exon1:c.C179T:p.A60V;10981425:exon1:c.C179T:p.A61V |
| *gene-UNC5B* | NW_013185654.1 | 10984601 | 10984601 | C | G | 10983917:exon1:c.C13G:p.P5A |
| *gene-UNC5B* | NW_013185654.1 | 10984828 | 10984828 | C | G | 10983917:exon1:c.C240G:p.D80E;10981425:exon2:c.C417G:p.D139E;10936064:exon5:c.C237G:p.D79E;10936064:exon6:c.C237G:p.D79E |
| *gene-UNC5B* | NW_013185654.1 | 10988246 | 10988246 | T | G | 10983917:exon3:c.T491G:p.V164G;10981425:exon4:c.T668G:p.V223G;10936064:exon7:c.T488G:p.V163G;10936064:exon8:c.T488G:p.V163G |
| *gene-UNC5B* | NW_013185654.1 | 10989233 | 10989233 | A | G | 10983917:exon4:c.A628G:p.M210V;10981425:exon5:c.A805G:p.M269V;10936064:exon8:c.A625G:p.M209V;10936064:exon9:c.A625G:p.M209V |
| *gene-UNC5B* | NW_013185654.1 | 10989517 | 10989517 | C | G | 10983917:exon5:c.C761G:p.T254R;10981425:exon6:c.C938G:p.T313R;10936064:exon9:c.C758G:p.T253R;10936064:exon10:c.C758G:p.T253R |
| *gene-UNC5B* | NW_013185654.1 | 10989868 | 10989868 | T | G | 10983917:exon6:c.T928G:p.C310G;10981425:exon7:c.T1105G:p.C369G;10936064:exon10:c.T925G:p.C309G;10936064:exon11:c.T925G:p.C309G |
| *gene-UNC5B* | NW_013185654.1 | 10991917 | 10991917 | C | G | 10981425:exon8:c.C1300G:p.R434G;10983917:exon8:c.C1156G:p.R386G;10936064:exon12:c.C1153G:p.R385G;10936064:exon13:c.C1153G:p.R385G |
| *gene-UNC5B* | NW_013185654.1 | 10991920 | 10991920 | T | G | 10981425:exon8:c.T1303G:p.C435G;10983917:exon8:c.T1159G:p.C387G;10936064:exon12:c.T1156G:p.C386G;10936064:exon13:c.T1156G:p.C386G |
| *gene-UNC5B* | NW_013185654.1 | 10992290 | 10992290 | A | C | 10981425:exon9:c.A1520C:p.D507A;10983917:exon9:c.A1376C:p.D459A;10936064:exon13:c.A1373C:p.D458A;10936064:exon14:c.A1373C:p.D458A |
| *gene-UNC5B* | NW_013185654.1 | 10992320 | 10992320 | A | C | 10981425:exon9:c.A1550C:p.Y517S;10983917:exon9:c.A1406C:p.Y469S;10936064:exon13:c.A1403C:p.Y468S;10936064:exon14:c.A1403C:p.Y468S |
| *gene-UNC5B* | NW_013185654.1 | 10992323 | 10992323 | A | C | 10981425:exon9:c.A1553C:p.N518T;10983917:exon9:c.A1409C:p.N470T;10936064:exon13:c.A1406C:p.N469T;10936064:exon14:c.A1406C:p.N469T |
| *gene-UNC5B* | NW_013185654.1 | 10992331 | 10992331 | A | C | 10981425:exon9:c.A1561C:p.T521P;10983917:exon9:c.A1417C:p.T473P;10936064:exon13:c.A1414C:p.T472P;10936064:exon14:c.A1414C:p.T472P |
| *gene-UNC5B* | NW_013185654.1 | 10992356 | 10992356 | A | C | 10981425:exon9:c.A1586C:p.H529P;10983917:exon9:c.A1442C:p.H481P;10936064:exon13:c.A1439C:p.H480P;10936064:exon14:c.A1439C:p.H480P |
| *gene-UNC5B* | NW_013185654.1 | 10992359 | 10992359 | A | C | 10981425:exon9:c.A1589C:p.D530A;10983917:exon9:c.A1445C:p.D482A;10936064:exon13:c.A1442C:p.D481A;10936064:exon14:c.A1442C:p.D481A |
| *gene-UNC5B* | NW_013185654.1 | 10992385 | 10992385 | C | G | 10981425:exon9:c.C1615G:p.P539A;10983917:exon9:c.C1471G:p.P491A;10936064:exon13:c.C1468G:p.P490A;10936064:exon14:c.C1468G:p.P490A |
| *gene-UNC5B* | NW_013185654.1 | 10992389 | 10992389 | G | C | 10981425:exon9:c.G1619C:p.S540T;10983917:exon9:c.G1475C:p.S492T;10936064:exon13:c.G1472C:p.S491T;10936064:exon14:c.G1472C:p.S491T |
| *gene-UNC5B* | NW_013185654.1 | 10992391 | 10992391 | A | C | 10981425:exon9:c.A1621C:p.T541P;10983917:exon9:c.A1477C:p.T493P;10936064:exon13:c.A1474C:p.T492P;10936064:exon14:c.A1474C:p.T492P |
| *gene-UNC5B* | NW_013185654.1 | 10992401 | 10992401 | A | C | 10981425:exon9:c.A1631C:p.Y544S;10983917:exon9:c.A1487C:p.Y496S;10936064:exon13:c.A1484C:p.Y495S;10936064:exon14:c.A1484C:p.Y495S |
| *gene-UNC5B* | NW_013185654.1 | 10992410 | 10992410 | A | G | 10981425:exon9:c.A1640G:p.D547G;10983917:exon9:c.A1496G:p.D499G;10936064:exon13:c.A1493G:p.D498G;10936064:exon14:c.A1493G:p.D498G |
| *gene-UNC5B* | NW_013185654.1 | 10992417 | 10992417 | T | G | 10981425:exon9:c.T1647G:p.S549R;10983917:exon9:c.T1503G:p.S501R;10936064:exon13:c.T1500G:p.S500R;10936064:exon14:c.T1500G:p.S500R |
| *gene-UNC5B* | NW_013185654.1 | 10992422 | 10992422 | A | G | 10981425:exon9:c.A1652G:p.D551G;10983917:exon9:c.A1508G:p.D503G;10936064:exon13:c.A1505G:p.D502G;10936064:exon14:c.A1505G:p.D502G |
| *gene-UNC5B* | NW_013185654.1 | 10992427 | 10992427 | C | G | 10981425:exon9:c.C1657G:p.H553D;10983917:exon9:c.C1513G:p.H505D;10936064:exon13:c.C1510G:p.H504D;10936064:exon14:c.C1510G:p.H504D |
| *gene-UNC5B* | NW_013185654.1 | 10992436 | 10992436 | A | G | 10981425:exon9:c.A1666G:p.N556D;10983917:exon9:c.A1522G:p.N508D;10936064:exon13:c.A1519G:p.N507D;10936064:exon14:c.A1519G:p.N507D |
| *gene-UNC5B* | NW_013185654.1 | 10992440 | 10992440 | T | G | 10981425:exon9:c.T1670G:p.V557G;10983917:exon9:c.T1526G:p.V509G;10936064:exon13:c.T1523G:p.V508G;10936064:exon14:c.T1523G:p.V508G |
| *gene-UNC5B* | NW_013185654.1 | 10992488 | 10992488 | A | G | 10981425:exon9:c.A1718G:p.E573G;10983917:exon9:c.A1574G:p.E525G;10936064:exon13:c.A1571G:p.E524G;10936064:exon14:c.A1571G:p.E524G |
| *gene-UNC5B* | NW_013185654.1 | 10992920 | 10992920 | G | A | 10981425:exon11:c.G1882A:p.E628K;10983917:exon11:c.G1738A:p.E580K;10936064:exon15:c.G1735A:p.E579K;10936064:exon16:c.G1735A:p.E579K |
| *gene-UNC5B* | NW_013185654.1 | 10993206 | 10993206 | T | G | 10981425:exon12:c.T2045G:p.V682G;10983917:exon12:c.T1901G:p.V634G;10936064:exon16:c.T1898G:p.V633G;10936064:exon17:c.T1898G:p.V633G |
| *gene-UNC5B* | NW_013185654.1 | 10993226 | 10993226 | A | G | 10981425:exon12:c.A2065G:p.T689A;10983917:exon12:c.A1921G:p.T641A;10936064:exon16:c.A1918G:p.T640A;10936064:exon17:c.A1918G:p.T640A |
| *gene-PNISR* | NW_013185673.1 | 8492089 | 8492089 | A | G | 8468474:exon12:c.A2443G:p.T815A |
| *gene-ARFGAP2* | NW_013185657.1 | 11004723 | 11004723 | A | G | 11001167:exon8:c.T806C:p.V269A |
| *gene-ARFGAP2* | NW_013185657.1 | 11009413 | 11009413 | G | A | 11001167:exon1:c.C151T:p.L51F |
| *gene-ARFGAP2* | NW_013185657.1 | 11009415 | 11009415 | G | A | 11001167:exon1:c.C149T:p.S50L |
| *gene-ARFGAP2* | NW_013185657.1 | 11009449 | 11009449 | A | G | 11001167:exon1:c.T115C:p.C39R |
| *gene-PPM1F* | NW_013185666.1 | 8510721 | 8510721 | A | G | 8498928:exon4:c.T227C:p.F76S |
| *gene-PACSIN3* | NW_013185657.1 | 11027175 | 11027175 | A | C | 11021099:exon4:c.T431G:p.V144G |
| *gene-PACSIN3* | NW_013185657.1 | 11028002 | 11028002 | T | C | 11021099:exon3:c.A118G:p.I40V |
| *gene-RASL11B* | NW_013185664.1 | 11075641 | 11075641 | C | G | 11075619:exon1:c.C22G:p.Q8E |
| *gene-RPAP3* | NW_013185658.1 | 11214724 | 11214724 | A | G | 11204902:exon11:c.A1309G:p.T437A |
| *gene-XRCC6BP1* | NW_013185658.1 | 11202569 | 11202569 | A | C | 11196926:exon1:c.T49G:p.Y17D |
| *gene-XRCC6BP1* | NW_013185658.1 | 11202577 | 11202577 | T | G | 11196926:exon1:c.A41C:p.D14A |
| *gene-XRCC6BP1* | NW_013185658.1 | 11202595 | 11202595 | T | C | 11196926:exon1:c.A23G:p.E8G |
| *gene-XRCC6BP1* | NW_013185658.1 | 11202598 | 11202598 | T | C | 11196926:exon1:c.A20G:p.E7G |
| *gene-SOD3* | NW_013185655.1 | 14209792 | 14209792 | G | A | 14209247:exon2:c.G52A:p.V18I;14208739:exon2:c.G52A:p.V19I |
| *gene-SPOCK3* | NW_013185671.1 | 8506552 | 8506552 | A | G | 8448114:exon3:c.T200C:p.L67P;8448114:exon4:c.T284C:p.L95P;8448113:exon6:c.T539C:p.L180P;8448113:exon7:c.T548C:p.L183P |
| *gene-OTUD1* | NW_013185661.1 | 11400076 | 11400076 | C | T | 11397677:exon2:c.G208A:p.A70T |
| *gene-OTUD1* | NW_013185661.1 | 11400118 | 11400118 | A | C | 11397677:exon2:c.T166G:p.F56V |
| *gene-OTUD1* | NW_013185661.1 | 11400139 | 11400139 | A | C | 11397677:exon2:c.T145G:p.Y49D |
| *gene-OTUD1* | NW_013185661.1 | 11400169 | 11400169 | A | C | 11397677:exon2:c.T115G:p.Y39D |
| *gene-OTUD1* | NW_013185661.1 | 11403892 | 11403892 | A | T | 11397677:exon1:c.T28A:p.L10I |
| *gene-SPON2* | NW_013185655.1 | 2063882 | 2063882 | G | T | 2062291:exon6:c.C1091A:p.P364H;2062292:exon7:c.C1049A:p.P350H;2062293:exon7:c.C920A:p.P307H;2062293:exon7:c.C920A:p.P307H;2062293:exon8:c.C920A:p.P307H;2062293:exon8:c.C920A:p.P307H |
| *gene-STOX1* | NW_013185654.1 | 11567516 | 11567516 | A | C | 11565820:exon3:c.T2337G:p.N779K;11565820:exon3:c.T2253G:p.N751K;11565820:exon3:c.T2193G:p.N731K;11565820:exon3:c.T2193G:p.N731K;11565820:exon4:c.T2193G:p.N731K |
| *gene-STOX1* | NW_013185654.1 | 11567649 | 11567649 | C | G | 11565820:exon3:c.G2204C:p.G735A;11565820:exon3:c.G2120C:p.G707A;11565820:exon3:c.G2060C:p.G687A;11565820:exon3:c.G2060C:p.G687A;11565820:exon4:c.G2060C:p.G687A |
| *gene-STOX1* | NW_013185654.1 | 11567698 | 11567698 | A | T | 11565820:exon3:c.T2155A:p.S719T;11565820:exon3:c.T2071A:p.S691T;11565820:exon3:c.T2011A:p.S671T;11565820:exon3:c.T2011A:p.S671T;11565820:exon4:c.T2011A:p.S671T |
| *gene-STOX1* | NW_013185654.1 | 11567730 | 11567730 | T | C | 11565820:exon3:c.A2123G:p.K708R;11565820:exon3:c.A2039G:p.K680R;11565820:exon3:c.A1979G:p.K660R;11565820:exon3:c.A1979G:p.K660R;11565820:exon4:c.A1979G:p.K660R |
| *gene-STOX1* | NW_013185654.1 | 11567856 | 11567856 | G | A | 11565820:exon3:c.C1997T:p.S666L;11565820:exon3:c.C1913T:p.S638L;11565820:exon3:c.C1853T:p.S618L;11565820:exon3:c.C1853T:p.S618L;11565820:exon4:c.C1853T:p.S618L |
| *gene-STOX1* | NW_013185654.1 | 11568010 | 11568010 | T | C | 11565820:exon3:c.A1843G:p.M615V;11565820:exon3:c.A1759G:p.M587V;11565820:exon3:c.A1699G:p.M567V;11565820:exon3:c.A1699G:p.M567V;11565820:exon4:c.A1699G:p.M567V |
| *gene-STOX1* | NW_013185654.1 | 11568027 | 11568027 | T | C | 11565820:exon3:c.A1826G:p.Q609R;11565820:exon3:c.A1742G:p.Q581R;11565820:exon3:c.A1682G:p.Q561R;11565820:exon3:c.A1682G:p.Q561R;11565820:exon4:c.A1682G:p.Q561R |
| *gene-STOX1* | NW_013185654.1 | 11568073 | 11568073 | T | C | 11565820:exon3:c.A1780G:p.K594E;11565820:exon3:c.A1696G:p.K566E;11565820:exon3:c.A1636G:p.K546E;11565820:exon3:c.A1636G:p.K546E;11565820:exon4:c.A1636G:p.K546E |
| *gene-STOX1* | NW_013185654.1 | 11568313 | 11568313 | C | T | 11565820:exon3:c.G1540A:p.G514S;11565820:exon3:c.G1456A:p.G486S;11565820:exon3:c.G1396A:p.G466S;11565820:exon3:c.G1396A:p.G466S;11565820:exon4:c.G1396A:p.G466S |
| *gene-STOX1* | NW_013185654.1 | 11568471 | 11568471 | C | A | 11565820:exon3:c.G1382T:p.R461L;11565820:exon3:c.G1298T:p.R433L;11565820:exon3:c.G1238T:p.R413L;11565820:exon3:c.G1238T:p.R413L;11565820:exon4:c.G1238T:p.R413L |
| *gene-STOX1* | NW_013185654.1 | 11568483 | 11568483 | G | A | 11565820:exon3:c.C1370T:p.S457L;11565820:exon3:c.C1286T:p.S429L;11565820:exon3:c.C1226T:p.S409L;11565820:exon3:c.C1226T:p.S409L;11565820:exon4:c.C1226T:p.S409L |
| *gene-STOX1* | NW_013185654.1 | 11568534 | 11568534 | C | T | 11565820:exon3:c.G1319A:p.G440D;11565820:exon3:c.G1235A:p.G412D;11565820:exon3:c.G1175A:p.G392D;11565820:exon3:c.G1175A:p.G392D;11565820:exon4:c.G1175A:p.G392D |
| *gene-STOX1* | NW_013185654.1 | 11568568 | 11568568 | A | G | 11565820:exon3:c.T1285C:p.W429R;11565820:exon3:c.T1201C:p.W401R;11565820:exon3:c.T1141C:p.W381R;11565820:exon3:c.T1141C:p.W381R;11565820:exon4:c.T1141C:p.W381R |
| *gene-STOX1* | NW_013185654.1 | 11568663 | 11568663 | G | A | 11565820:exon3:c.C1190T:p.S397L;11565820:exon3:c.C1106T:p.S369L;11565820:exon3:c.C1046T:p.S349L;11565820:exon3:c.C1046T:p.S349L;11565820:exon4:c.C1046T:p.S349L |
| *gene-STOX1* | NW_013185654.1 | 11568667 | 11568667 | T | C | 11565820:exon3:c.A1186G:p.I396V;11565820:exon3:c.A1102G:p.I368V;11565820:exon3:c.A1042G:p.I348V;11565820:exon3:c.A1042G:p.I348V;11565820:exon4:c.A1042G:p.I348V |
| *gene-STOX1* | NW_013185654.1 | 11569060 | 11569060 | C | G | 11565820:exon3:c.G793C:p.E265Q;11565820:exon3:c.G709C:p.E237Q;11565820:exon3:c.G649C:p.E217Q;11565820:exon3:c.G649C:p.E217Q;11565820:exon4:c.G649C:p.E217Q |
| *gene-STOX1* | NW_013185654.1 | 11569198 | 11569198 | C | G | 11565820:exon3:c.G655C:p.E219Q;11565820:exon3:c.G571C:p.E191Q;11565820:exon3:c.G511C:p.E171Q;11565820:exon3:c.G511C:p.E171Q;11565820:exon4:c.G511C:p.E171Q |
| *gene-STOX1* | NW_013185654.1 | 11569222 | 11569222 | G | A | 11565820:exon3:c.C631T:p.R211C;11565820:exon3:c.C547T:p.R183C;11565820:exon3:c.C487T:p.R163C;11565820:exon3:c.C487T:p.R163C;11565820:exon4:c.C487T:p.R163C |
| *gene-STOX1* | NW_013185654.1 | 11579799 | 11579799 | A | G | 11565820:exon1:c.T101C:p.F34S |
| *gene-STOX1* | NW_013185654.1 | 11579841 | 11579841 | A | T | 11565820:exon1:c.T59A:p.L20H |
| *gene-STOX1* | NW_013185654.1 | 11579842 | 11579842 | G | C | 11565820:exon1:c.C58G:p.L20V |
| *gene-STOX1* | NW_013185654.1 | 11579845 | 11579845 | G | C | 11565820:exon1:c.C55G:p.Q19E |
| *gene-STOX1* | NW_013185654.1 | 11567355 | 11567355 | C | T | 11565820:exon3:c.G2498A:p.C833Y;11565820:exon3:c.G2414A:p.C805Y;11565820:exon3:c.G2354A:p.C785Y;11565820:exon3:c.G2354A:p.C785Y;11565820:exon4:c.G2354A:p.C785Y |
| *gene-CCAR1* | NW_013185654.1 | 11592553 | 11592553 | C | T | 11590495:exon24:c.G3406A:p.V1136I |
| *gene-CCAR1* | NW_013185654.1 | 11606287 | 11606287 | A | C | 11590495:exon9:c.T1187G:p.V396G |
| *gene-CCAR1* | NW_013185654.1 | 11611585 | 11611585 | C | T | 11590495:exon2:c.G166A:p.V56M |
| *gene-CCAR1* | NW_013185654.1 | 11611593 | 11611593 | C | T | 11590495:exon2:c.G158A:p.R53H |
| *gene-CCAR1* | NW_013185654.1 | 11612945 | 11612945 | T | C | 11590495:exon1:c.A40G:p.S14G |
| *gene-CCAR1* | NW_013185654.1 | 11612968 | 11612968 | G | A | 11590495:exon1:c.C17T:p.T6M |
| *gene-TACC3* | NW_013185655.1 | 3131757 | 3131757 | C | T | 3128268:exon3:c.C329T:p.P110L |
| *gene-FAM110C* | NW_013185662.1 | 12822608 | 12822608 | T | C | 12817335:exon1:c.A295G:p.M99V |
| *gene-FAM110C* | NW_013185662.1 | 12822721 | 12822721 | G | A | 12817335:exon1:c.C182T:p.S61L |
| *gene-FAM110C* | NW_013185662.1 | 12822740 | 12822740 | T | C | 12817335:exon1:c.A163G:p.K55E |
| *gene-FAM110C* | NW_013185662.1 | 12822787 | 12822787 | G | A | 12817335:exon1:c.C116T:p.P39L |
| *gene-FAM110C* | NW_013185662.1 | 12822856 | 12822856 | G | T | 12817335:exon1:c.C47A:p.A16E |
| *gene-FAM110C* | NW_013185662.1 | 12822890 | 12822890 | T | G | 12817335:exon1:c.A13C:p.T5P |
| *gene-TDP1* | NW_013185655.1 | 20773443 | 20773443 | C | T | 20729744:exon2:c.G196A:p.G66S;20729744:exon2:c.G196A:p.G66S;20729743:exon2:c.G196A:p.G66S;20729743:exon2:c.G196A:p.G66S;20729743:exon2:c.G196A:p.G66S |
| *gene-SOD3* | NW_013185655.1 | 14209960 | 14209960 | G | A | 14209247:exon2:c.G220A:p.D74N;14208739:exon2:c.G220A:p.D75N |
| *gene-SOD3* | NW_013185655.1 | 14210059 | 14210059 | A | G | 14209247:exon2:c.A319G:p.T107A;14208739:exon2:c.A319G:p.T108A |
| *gene-SOD3* | NW_013185655.1 | 14210195 | 14210195 | A | G | 14209247:exon2:c.A455G:p.K152R;14208739:exon2:c.A455G:p.K153R |
| *gene-SOD3* | NW_013185655.1 | 14210195 | 14210195 | A | G | 14209247:exon2:c.A455G:p.K152R;14208739:exon2:c.A455G:p.K153R |
| *gene-SOD3* | NW_013185655.1 | 14210416 | 14210416 | G | T | 14209247:exon2:c.G676T:p.A226S;14208739:exon2:c.G676T:p.A227S |
| *gene-TINAG* | NW_013185662.1 | 8501742 | 8501742 | C | T | 8501730:exon11:c.G1393A:p.A465T |
| *gene-PI4K2B* | NW_013185655.1 | 14358356 | 14358356 | C | T | 14356254:exon2:c.C131T:p.T44M;14357412:exon2:c.C59T:p.T20M |
| *gene-PI4K2B* | NW_013185655.1 | 14358466 | 14358466 | G | A | 14356254:exon2:c.G241A:p.A81T;14357412:exon2:c.G169A:p.A57T |
| *gene-PI4K2B* | NW_013185655.1 | 14372374 | 14372374 | G | A | 14356254:exon10:c.G1231A:p.A411T;14357412:exon10:c.G1159A:p.A387T |
| *gene-TMEM251* | NW_013185655.1 | 19192312 | 19192312 | G | A | 19191640:exon2:c.C383T:p.T128M |
| *gene-ZCCHC4* | NW_013185655.1 | 14376832 | 14376832 | C | T | 14375232:exon2:c.C241T:p.P81S |
| *gene-ZCCHC4* | NW_013185655.1 | 14377587 | 14377587 | C | G | 14375232:exon3:c.C328G:p.Q110E |
| *gene-ZCCHC4* | NW_013185655.1 | 14383713 | 14383713 | G | A | 14375232:exon11:c.G1247A:p.S416N |
| *gene-ZCCHC4* | NW_013185655.1 | 14385519 | 14385519 | G | C | 14375232:exon12:c.G1386C:p.R462S |
| *gene-ZCCHC4* | NW_013185655.1 | 14385530 | 14385530 | A | G | 14375232:exon12:c.A1397G:p.K466R |
| *gene-ANAPC4* | NW_013185655.1 | 14387159 | 14387159 | A | G | 14387143:exon1:c.A1G:p.M1V |
| *gene-ANAPC4* | NW_013185655.1 | 14387169 | 14387169 | A | C | 14387143:exon1:c.A11C:p.D4A |
| *gene-ANAPC4* | NW_013185655.1 | 14399466 | 14399466 | C | A | 14387873:exon20:c.C1222A:p.P408T;14387873:exon20:c.C1222A:p.P408T |
| *gene-ANAPC4* | NW_013185655.1 | 14402729 | 14402729 | G | A | 14387873:exon25:c.G1717A:p.G573R;14387873:exon25:c.G1717A:p.G573R |
| *gene-TINAG* | NW_013185662.1 | 8523078 | 8523078 | T | C | 8501730:exon8:c.A1102G:p.N368D |
| *gene-NCOA4* | NW_013185654.1 | 17018171 | 17018171 | C | T | 17014481:exon3:c.C229T:p.R77C |
| *gene-NCOA4* | NW_013185654.1 | 17018951 | 17018951 | G | A | 17014481:exon5:c.G416A:p.G139E |
| *gene-NCOA4* | NW_013185654.1 | 17020973 | 17020973 | G | T | 17014481:exon8:c.G835T:p.A279S |
| *gene-NCOA4* | NW_013185654.1 | 17021492 | 17021492 | A | G | 17014481:exon8:c.A1354G:p.I452V |
| *gene-NCOA4* | NW_013185654.1 | 17021703 | 17021703 | G | C | 17014481:exon8:c.G1565C:p.S522T |
| *gene-TMEM87A* | NW_013185657.1 | 8394663 | 8394663 | A | C | 8379829:exon12:c.A1153C:p.M385L |
| *gene-UBR7* | NW_013185655.1 | 19177619 | 19177619 | A | C | 19173837:exon7:c.T761G:p.V254G |
| *gene-UBR7* | NW_013185655.1 | 19178212 | 19178212 | T | C | 19173837:exon6:c.A680G:p.H227R |
| *gene-UBR7* | NW_013185655.1 | 19183626 | 19183626 | A | G | 19173837:exon2:c.T245C:p.L82P |
| *gene-UBR7* | NW_013185655.1 | 19183666 | 19183666 | A | T | 19173837:exon2:c.T205A:p.W69R |
| *gene-UBR7* | NW_013185655.1 | 19183770 | 19183770 | A | C | 19173837:exon2:c.T101G:p.V34G |
| *gene-UBR7* | NW_013185655.1 | 19183786 | 19183786 | A | T | 19173837:exon2:c.T85A:p.C29S |
| *gene-UBR7* | NW_013185655.1 | 19183797 | 19183797 | C | A | 19173837:exon2:c.G74T:p.R25L |
| *gene-UBR7* | NW_013185655.1 | 19185934 | 19185934 | G | A | 19173837:exon1:c.C70T:p.R24C |
| *gene-UBR7* | NW_013185655.1 | 19185964 | 19185964 | A | C | 19173837:exon1:c.T40G:p.L14V |
| *gene-UBR7* | NW_013185655.1 | 19185969 | 19185969 | C | T | 19173837:exon1:c.G35A:p.R12H |
| *gene-TWISTNB* | NW_013185661.1 | 1316720 | 1316720 | T | C | 1315539:exon3:c.A502G:p.T168A |
| *gene-UBR7* | NW_013185655.1 | 19185999 | 19185999 | G | A | 19173837:exon1:c.C5T:p.A2V |
| *gene-TMEM251* | NW_013185655.1 | 19194911 | 19194911 | C | G | 19191640:exon1:c.G160C:p.E54Q |
| *gene-TMEM251* | NW_013185655.1 | 19194965 | 19194965 | C | T | 19191640:exon1:c.G106A:p.E36K |
| *gene-TMEM251* | NW_013185655.1 | 19194980 | 19194980 | A | G | 19191640:exon1:c.T91C:p.W31R |
| *gene-TMEM251* | NW_013185655.1 | 19194999 | 19194999 | T | G | 19191640:exon1:c.A72C:p.E24D |
| *gene-TMEM251* | NW_013185655.1 | 19195066 | 19195066 | G | A | 19191640:exon1:c.C5T:p.T2I |
| *gene-UNC5B* | NW_013185654.1 | 10981586 | 10981586 | T | C | 10981425:exon1:c.T95C:p.V32A;10981425:exon1:c.T95C:p.V33A |
| *gene-KCNK13* | NW_013185655.1 | 20656793 | 20656793 | A | G | 20652177:exon2:c.T614C:p.M205T |
| *gene-TDP1* | NW_013185655.1 | 20737107 | 20737107 | C | T | 20729744:exon15:c.G1679A:p.G560E;20729744:exon15:c.G1679A:p.G560E;20729743:exon15:c.G1679A:p.G560E;20729743:exon15:c.G1679A:p.G560E;20729743:exon15:c.G1679A:p.G560E |
| *gene-TDP1* | NW_013185655.1 | 20737114 | 20737114 | G | A | 20729744:exon15:c.C1672T:p.L558F;20729744:exon15:c.C1672T:p.L558F;20729743:exon15:c.C1672T:p.L558F;20729743:exon15:c.C1672T:p.L558F;20729743:exon15:c.C1672T:p.L558F |
| *gene-TDP1* | NW_013185655.1 | 20759333 | 20759333 | A | G | 20729744:exon10:c.T1160C:p.L387S;20729744:exon10:c.T1160C:p.L387S;20729743:exon10:c.T1160C:p.L387S;20729743:exon10:c.T1160C:p.L387S;20729743:exon10:c.T1160C:p.L387S |
| *gene-TDP1* | NW_013185655.1 | 20762996 | 20762996 | C | T | 20729744:exon8:c.G972A:p.M324I;20729744:exon8:c.G972A:p.M324I;20729743:exon8:c.G972A:p.M324I;20729743:exon8:c.G972A:p.M324I;20729743:exon8:c.G972A:p.M324I |
| *gene-TDP1* | NW_013185655.1 | 20768880 | 20768880 | T | C | 20729744:exon5:c.A695G:p.E232G;20729744:exon5:c.A695G:p.E232G;20729743:exon5:c.A695G:p.E232G;20729743:exon5:c.A695G:p.E232G;20729743:exon5:c.A695G:p.E232G |
| *gene-TDP1* | NW_013185655.1 | 20768883 | 20768883 | G | T | 20729744:exon5:c.C692A:p.A231D;20729744:exon5:c.C692A:p.A231D;20729743:exon5:c.C692A:p.A231D;20729743:exon5:c.C692A:p.A231D;20729743:exon5:c.C692A:p.A231D |
| *gene-TDP1* | NW_013185655.1 | 20773208 | 20773208 | C | T | 20729744:exon2:c.G431A:p.S144N;20729744:exon2:c.G431A:p.S144N;20729743:exon2:c.G431A:p.S144N;20729743:exon2:c.G431A:p.S144N;20729743:exon2:c.G431A:p.S144N |
| *gene-TDP1* | NW_013185655.1 | 20773212 | 20773212 | G | A | 20729744:exon2:c.C427T:p.P143S;20729744:exon2:c.C427T:p.P143S;20729743:exon2:c.C427T:p.P143S;20729743:exon2:c.C427T:p.P143S;20729743:exon2:c.C427T:p.P143S |
| *gene-TDP1* | NW_013185655.1 | 20773287 | 20773287 | G | A | 20729744:exon2:c.C352T:p.P118S;20729744:exon2:c.C352T:p.P118S;20729743:exon2:c.C352T:p.P118S;20729743:exon2:c.C352T:p.P118S;20729743:exon2:c.C352T:p.P118S |
| *gene-TDP1* | NW_013185655.1 | 20773307 | 20773307 | T | C | 20729744:exon2:c.A332G:p.K111R;20729744:exon2:c.A332G:p.K111R;20729743:exon2:c.A332G:p.K111R;20729743:exon2:c.A332G:p.K111R;20729743:exon2:c.A332G:p.K111R |
| *gene-TDP1* | NW_013185655.1 | 20773315 | 20773315 | T | G | 20729744:exon2:c.A324C:p.E108D;20729744:exon2:c.A324C:p.E108D;20729743:exon2:c.A324C:p.E108D;20729743:exon2:c.A324C:p.E108D;20729743:exon2:c.A324C:p.E108D |
| *gene-USP54* | NW_013185654.1 | 23949617 | 23949617 | C | G | 23849739:exon24:c.C4850G:p.S1617W |
| *gene-EFCAB11* | NW_013185655.1 | 20774122 | 20774122 | C | A | 20774062:exon1:c.C19A:p.P7T |
| *gene-VPS39* | NW_013185657.1 | 8422948 | 8422948 | G | C | 8406466:exon19:c.G1983C:p.K661N;8406464:exon20:c.G2016C:p.K673N |
| *gene-VTI1A* | NW_013185669.1 | 7858361 | 7858361 | A | G | 7693502:exon9:c.A692G:p.N231S |
| *gene-AP3M1* | NW_013185654.1 | 23172043 | 23172043 | C | T | 23167673:exon5:c.C649T:p.P217S |
| *gene-AP3M1* | NW_013185654.1 | 23172044 | 23172044 | C | T | 23167673:exon5:c.C650T:p.P217L |
| *gene-AP3M1* | NW_013185654.1 | 23172050 | 23172050 | T | C | 23167673:exon5:c.T656C:p.F219S |
| *gene-AP3M1* | NW_013185654.1 | 23172056 | 23172056 | A | T | 23167673:exon5:c.A662T:p.Y221F |
| *gene-PLAU* | NW_013185654.1 | 23290090 | 23290090 | C | T | 23288577:exon11:c.G1276A:p.A426T |
| *gene-PLAU* | NW_013185654.1 | 23290125 | 23290125 | T | C | 23288577:exon11:c.A1241G:p.N414S |
| *gene-PLAU* | NW_013185654.1 | 23291120 | 23291120 | C | T | 23288577:exon10:c.G1087A:p.A363T |
| *gene-PLAU* | NW_013185654.1 | 23292607 | 23292607 | T | C | 23288577:exon8:c.A739G:p.M247V |
| *gene-PLAU* | NW_013185654.1 | 23293387 | 23293387 | C | T | 23288577:exon7:c.G581A:p.R194H |
| *gene-XRCC6BP1* | NW_013185658.1 | 11197193 | 11197193 | C | T | 11196926:exon6:c.G644A:p.S215N |
| *gene-NDST2* | NW_013185654.1 | 23595473 | 23595473 | T | G | 23595035:exon1:c.T32G:p.V11G |
| *gene-NDST2* | NW_013185654.1 | 23595593 | 23595593 | C | A | 23595035:exon1:c.C152A:p.P51H |
| *gene-NDST2* | NW_013185654.1 | 23612262 | 23612262 | A | C | 23595035:exon8:c.A1847C:p.H616P |
| *gene-NDST2* | NW_013185654.1 | 23616053 | 23616053 | T | G | 23595035:exon12:c.T2372G:p.L791W |
| *gene-ZCCHC4* | NW_013185655.1 | 14375278 | 14375278 | G | A | 14375232:exon1:c.G46A:p.G16R |
| *gene-USP54* | NW_013185654.1 | 23934757 | 23934757 | G | A | 23849739:exon16:c.G2206A:p.G736R |
| *gene-USP54* | NW_013185654.1 | 23934758 | 23934758 | G | A | 23849739:exon16:c.G2207A:p.G736E |
| *gene-USP54* | NW_013185654.1 | 23934809 | 23934809 | T | G | 23849739:exon16:c.T2258G:p.V753G |
| *gene-USP54* | NW_013185654.1 | 23938016 | 23938016 | G | T | 23849739:exon17:c.G2467T:p.A823S |
| *gene-USP54* | NW_013185654.1 | 23948434 | 23948434 | G | C | 23849739:exon22:c.G4445C:p.S1482T |
| *gene-ZNF408* | NW_013185657.1 | 10759159 | 10759159 | G | C | 10758467:exon1:c.G134C:p.G45A |

**Table S2** Reverse transcription reaction system

| Components | Volume |
| --- | --- |
| 5× Reaction Buffer | 4 μL |
| Oligo (dT)_18_ Primer (100 μM) | 0.5 μL |
| Random Hexamer Primer (100 μM) | 0.5 μL |
| Servicebio^®^ RT Enzyme Mix | 1 μL |
| Total RNA^*^ | 10 μL |
| RNase free water | Make up volume to 20 μL |

**Table S3** Primer sequences for gene expression analysis

| Gene name | Primer sequence | Length (bp) |
| --- | --- | --- |
| *GAPDH* | F: GGTGCTAAGCGTGTCATCATCTC | 179 |
|  | R: AGACCCTCCACGATGCCAAA |  |
| *PACSIN3* | F: CTGGCGAAGATTCGGATAAGAT | 132 |
|  | R: TCTTCTTCACCCAGGGCTTCT |  |
| *KIF26B* | F: TGGCTATTCAGGCTCATCAGTACC | 159 |
|  | R: AGGGTTTATCTGCATTGTAGCGG |  |
| *CAPN3* | F: TGCCTACCCACATACAACAACC | 178 |
|  | R: CATAGAACTCTGTCACTCCTCCTGT |  |
| *PLAU* | F: GTGAGCACGATGGCAGGATG | 150 |
|  | R: AACGACTTTTGGTGAACACTGCAT |  |

**Table S4** qRT-PCR system

| Components | Volume |
| --- | --- |
| 2× qRT-PCR Mix | 7.5 μL |
| 2.5 μM Gene Primers (forward + reverse) | 1.5 μL |
| Reverse-transcribed Product (cDNA) | 2.0 μL |
| Nuclease-free Water | 4.0 μL |

**Table S5** qRT-PCR conditions

| Stage 1 | Stage 2 (40 cycles) | Stage 3 (Melting Curve) |
| --- | --- | --- |
| 95℃, 30 s  Pre-denaturation | 95℃, 15 s Denaturation | 65℃→95℃ |
|  | 60℃, 30 s Annealing/Extension | Fluorescence signal collected after every 0.5°C increase in temperature |
